# The Extracytoplasmic Linker Peptide of the Sensor Protein SaeS Tunes the Kinase Activity Required for Staphylococcal Virulence in Response to Host Signals

**DOI:** 10.1371/journal.ppat.1004799

**Published:** 2015-04-07

**Authors:** Qian Liu, Hoonsik Cho, Won-Sik Yeo, Taeok Bae

**Affiliations:** Department of Microbiology and Immunology, Indiana University School of Medicine-Northwest,Gary, Indiana, United States of America; Harbor-UCLA Medical Center, UNITED STATES

## Abstract

Bacterial pathogens often employ two-component systems (TCSs), typically consisting of a sensor kinase and a response regulator, to control expression of a set of virulence genes in response to changing host environments. In *Staphylococcus aureus*, the SaeRS TCS is essential for in vivo survival of the bacterium. The intramembrane-sensing histidine kinase SaeS contains, along with a C-terminal kinase domain, a simple N-terminal domain composed of two transmembrane helices and a nine amino acid-long extracytoplasmic linker peptide. As a molecular switch, SaeS maintains low but significant basal kinase activity and increases its kinase activity in response to inducing signals such as human neutrophil peptide 1 (HNP1). Here we show that the linker peptide of SaeS controls SaeS’s basal kinase activity and that the amino acid sequence of the linker peptide is highly optimized for its function. Without the linker peptide, SaeS displays aberrantly elevated kinase activity even in the absence of the inducing signal, and does not respond to HNP1. Moreover, SaeS variants with alanine substitution of the linker peptide amino acids exhibit altered basal kinase activity and/or irresponsiveness to HNP1. Biochemical assays reveal that those SaeS variants have altered autokinase and phosphotransferase activities. Finally, animal experiments demonstrate that the linker peptide-mediated fine tuning of SaeS kinase activity is critical for survival of the pathogen. Our results indicate that the function of the linker peptide in SaeS is a highly evolved feature with very optimized amino acid sequences, and we propose that, in other SaeS-like intramembrane sensing histidine kinases, the extracytoplasmic linker peptides actively fine-control their kinases.

## Author Summary

A bacterial pathogen *Staphylococcus aureus* uses the SaeRS two-component system to control the production of multiple toxins, resulting in a wide range of diseases in human. The sensor kinase SaeS is a member of the intramembrane-sensing histidine kinases (IM-HKs) that lacks a sensory domain and harbors a simple N-terminal domain with two transmembrane helices and a short linker peptide. It’s been considered that the linker peptide of IM-HKs transmits the external signals into the cytoplasmic catalytic domain to control the HK’s kinase activity. However, it is unclear how the external signal input propagates through the linker to modulate the kinase activity of HKs. Here we show that the linker peptide of SaeS is critical in maintaining the basal kinase activity and functions as a part of a “tripwire” to jumpstart the activation of the SaeRS system upon exposure to the specific host signals. We establish that a single amino acid substitution of the linker peptide alters SaeS’s kinase activity, resulting in different expression levels of the SaeR-activated genes and alteration of the bacterial virulence in mice. Our study provides new molecular insights into how the pathogenic bacterium utilizes the simple protein domain to control its disease-causing potentials in response to host immune signals.

## Introduction

Two-component signal transduction systems (TCSs) are a major sensory-regulatory mechanism utilized by most bacteria to monitor and respond to various environmental stimuli such as nutrient concentrations, ionic strength, and antimicrobial substances [1,2]. A simple TCS consists of two proteins: a sensor histidine kinase (HK) and a response regulator (RR). Upon sensing a cognate ligand, the HK autophosphorylates its conserved histidine residue; then the phosphoryl group is transferred to the aspartate residue of its cognate RR. The phosphorylated RR carries out the adaptive response to the environmental signal, typically by altering gene expression acting as a transcription regulator [3,4,5]. Although the downstream signaling pathway controlled by the phosphorylated RR is well understood, in most TCSs, the signal sensing step is not clearly defined.

Typically, the N-terminus of HKs contains a large extracytoplasmic domain between two transmembrane helices, which is expected to bind cognate signals. However, a subset of HKs, classified as intramembrane-sensing HKs (IM-HKs), lack the extracytoplasmic domain, and their transmembrane helices are connected by a short linker peptide (<25 amino acids), which is too small to function as a signal binding domain [6]. IM-HKs are known to require additional component(s) for the signal sensing. For example, BceS and LiaS, the IM-HKs involved in sensing cell wall targeting antimicrobials, need an ABC transporter or a membrane protein to respond to their cognate signals [7,8], indicating that the N-terminal region of IM-HKs is involved in signal transfer, not signal sensing [9]. However, it is not clearly defined how the N-terminal domain transfers the signal to modulate the kinase activity of IM-HKs.

In *Staphylococcus aureus*, an important Gram-positive human pathogen, the SaeRS TCS detects the human neutrophil peptides (HNPs) and controls the production of over 20 important virulence factors including alpha-hemolysin (Hla), coagulase (Coa), leukocidins, fibronectin binding proteins (FnBPs), and proteases [10,11,12,13,14,15]. This TCS consists of an IM-HK SaeS, the response regulator SaeR, and two auxiliary proteins SaeP and SaeQ. The auxiliary proteins SaeP and SaeQ are a lipoprotein and a membrane protein, respectively, and their expression is induced by phosphorylated SaeR (i.e., autoinduction) from the P1 promoter. Upon being induced, the two proteins bind to SaeS and convert SaeS from a kinase to a phosphatase, returning the SaeRS TCS to the ground state [16,17]. However, neither protein is involved in sensing HNPs.

Two distinct groups of *sae* targets are known: low affinity (or class I) (e.g., coagulase [*coa*] and the P1 promoter of the *sae* operon) and high affinity (or class II) targets (e.g., alpha-hemolysin [*hla*]) [18,19]. The *coa* and the P1 promoters have two SaeR binding sites, and their transcription requires the induction of the SaeRS TCS [19]. On the other hand, the *hla* promoter has one SaeR binding site and is transcribed constitutively regardless of the activation of the SaeRS TCS. In fact, the *hla* transcription level is not significantly increased upon the HNP1-mediated induction of the SaeRS TCS [16,17]. Therefore, as a molecular switch, SaeS requires the following properties: 1) Its basal kinase activity should be high enough to support the transcription of the high affinity targets (e.g., *hla*) but low enough to suppress the expression of low affinity targets (e.g., *coa* and *saePQ*); 2) Its kinase activity should be increased in response to inducing signals.

**Fig 1:**
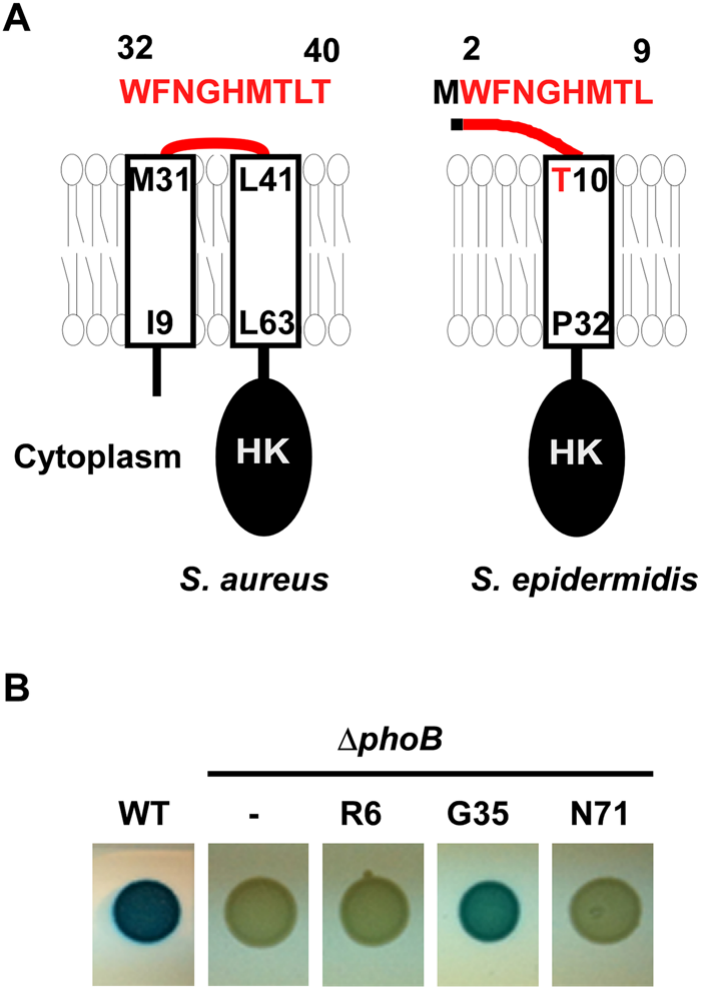
The topology of the N-terminal domain of SaeS. (A) The predicted topologies of the N-terminal domain of SaeS in *S*. *aureus* and *S*. *epidermidis*. Transmembrane helices were shown as rectangles, where the terminal amino acids and their positions are indicated. The amino acid sequence of the linker peptide is shown in red above the topology model with the amino acid positions. HK, histidine kinase domain. (B) The confirmation of surface exposure of Gly35 in the linker peptide. The *phoB* gene was inserted into *saeS* in the single copy plasmid pCL-*saeRS* at the position Arg6 (R6), Gly35 (G35), and Asn71 (N71); then the plasmids were inserted into the *phoB*-deletion mutant of *S*. *aureus* strain Newman (NMΔ*phoB*). The test strains were grown on a tryptic soy agar containing XP (5-Bromo-4-chloro-3-indolyl phosphate). WT, wild type; *ΔphoB*, *phoB-* deletion mutant;-, no *phoB*-fusion.

The N-terminal domain of SaeS is predicted to be composed of two membrane helices connected by an extracytoplasmic linker peptide (hereafter linker peptide) of nine amino acids (Fig 1A and S1 Fig), although the boundary amino acids of the linker peptide have not been experimentally verified. The transmembrane helices (TMs) appear to be critical for controlling the SaeS kinase activity: The L18P mutation in the first TM transforms SaeS into a constitutively active kinase while the I9Q mutation or the double mutation of I9Q/L63Q in the first or the second TM reduces the basal kinase activity [20,21]. The linker peptide is also known to be important for the kinase activity. When the linker peptide was shortened by deletion mutagenesis, the resulting mutant SaeS proteins showed a higher (Δ35–37) or a lower (SaeS Δ34–37) basal kinase activity [21]. Flack et al have recently reported that the three conserved amino acids M31, W32, and F33 are critical for maintaining the basal kinase activity of SaeS. However, it is still not clear how the N-terminal domain, in particular the linker peptide, controls the kinase activity of SaeS in the absence or presence of the inducing signals such as HNP1.

To investigate the role of the N-terminal domain of SaeS in controlling its kinase activity, we dissected the domain by a comprehensive mutagenesis approach. Our findings suggest that the transmembrane helices and the linker peptide act as a coherent unit and confer responsiveness to specific host signals. In particular, the linker peptide restrains the kinase activity of SaeS and is required for the induction of SaeS’s kinase activity by HNP1. Moreover, the amino acid sequence of the linker peptide is highly optimized to precisely control the kinase activity of SaeS and staphylococcal virulence in host.

## Results

### The SaeS Topology

Sequence analysis (SMART, http://smart.embl-heidelberg.de/) predicted that the N-terminal domain of SaeS consists of two transmembrane helices (a.a. 9–31, a.a. 41–63) connected by a nine amino acid-linker peptide (a.a. 32–40 in Fig 1A) [20]. To examine the predicted topology of SaeS, we fused the *phoB* gene, encoding staphylococcal alkaline phosphatase, to *saeS* at R6, G35, and N71 positions, and assessed the alkaline phosphatase activity. Since alkaline phosphatase is active only in the extracytoplasmic environment, the activity of the enzyme in a fusion protein can reveal the topology of membrane protein [22]. As shown in Fig 1B, the G35 fusion showed significant alkaline phosphatase activity whereas the R6 and N71 fusions did not, demonstrating that G35 of SaeS is exposed to the extracytoplasmic environment, as the topology model suggested.

### The N-terminal Domain of SaeS Controls the Basal Expression Level and Response to HNP1

To measure the kinase activity of SaeS, we used a GFP reporter for two promoters: the coagulase promoter (P*coa*) and the alpha-hemolysin promoter (P*hla*). As a low affinity target of the phosphorylated SaeR (SaeR-P), P*coa* can sensitively detect the increase of kinase activity of SaeS; however, it cannot distinguish a small decrease of kinase activity from a large decrease (S2 Fig). On the other hand, as a high affinity target, P*hla* is rather insensitive to the increase of SaeS’s kinase activity; however, it can detect a large decrease of the kinase activity of SaeS (S2 Fig). In our mutagenesis study, we used a *sae* deletion mutant complemented with a single copy plasmid where SaeR and SaeS are produced from their native promoter P3. By using this strain, we eliminated any artifacts from multi-copy plasmids, preserved the stoichiometry of SaeR and SaeS, and avoided complications from the expression of SaeP and SaeQ, which reduce the overall kinase activity of SaeS.

**Fig 2:**
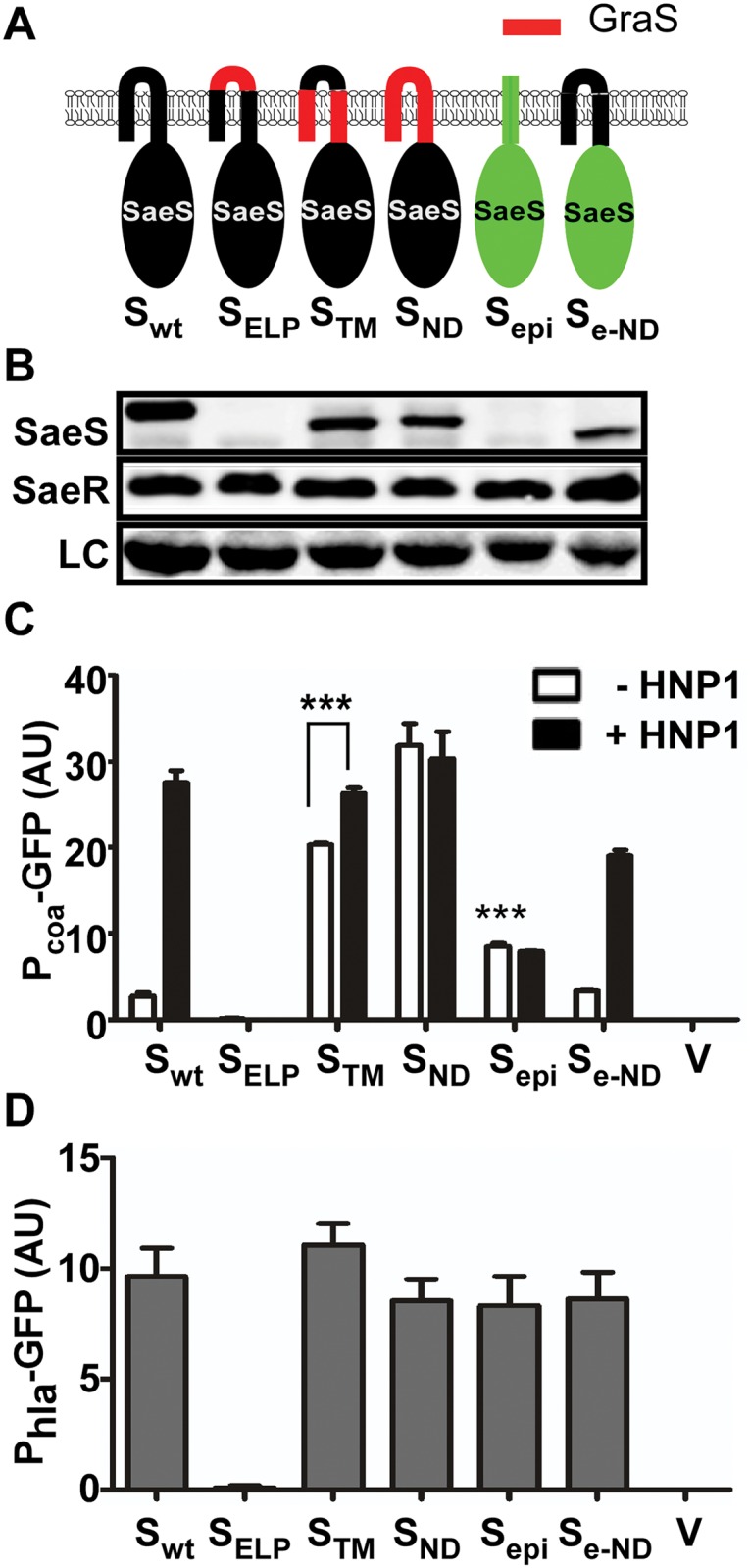
The N-terminal domain of SaeS controls the basal expression level and response to HNP1. (A) A diagram for the hybrid SaeS mutants. SaeS sequence is indicated in black while the sequence of GraS, an intramembrane sensing kinase in *S*. *aureus*, is in red. The sequence of *S*. *epidermidis* SaeS is shown in green. wt, wild type; ELP, extracytoplasmic linker peptide; TM, transmembrane helices; ND, N-terminal domain; epi, *S*. *epidermidis*; e-ND, *S*. *epidermidis* SaeS with *S*. *aureus* N-terminal domain. (B) Expression of the hybrid SaeS proteins analyzed by Western blot. Test strains were grown until exponential growth phase, and an equal number of cells were used for the analysis. As a loading control (LC), a nonspecific protein band observed across the test strains was used. (C) The effect of the mutations on the transcription of the low affinity target P*coa*, the coagulase promoter. Cells were grown until exponential growth phase. For induction of the SaeRS TCS, HNP1 (5 μg/ml) was added and the culture was further incubated for 2 h. The GFP expression was normalized with OD_600_. The experiments were repeated at least three times with similar results observed. Error bars represents the standard error of the mean. Unless indicated otherwise, GFP expression was compared with that of the strain carrying wild type SaeS, and statistical significance was assessed by unpaired, two-tailed student’s t-test. ***, p < 0.001; AU, arbitrary unit; V, vector control. (D) The effect of the mutations on the transcription of the high affinity target P*hla*, the alpha-hemolysin promoter. Cells at exponential growth phase were used. Error bars indicate the standard error of the mean.

First, we replaced either a part of or the entire N-terminal domain of SaeS with the corresponding sequence of GraS, another IM-HK in *S*. *aureus* (Fig 2A). When the linker peptide was replaced with that of GraS, neither the protein nor the kinase activity was detected (S_ELP_ in Fig 2B–2D), indicating that the hybrid SaeS is unstable. When the regions of the two transmembrane helices or the entire N-terminal domain was replaced with those from GraS, the promoter activity of P*coa* was increased 6–8 times with poor response to HNP1 (S_TM_ and S_ND_ in Fig 2C). These results suggest that the N-terminal domain is critical in keeping the basal kinase activity low and responding to HNP1.

The SaeS homolog in *S*. *epidermidis* (SaeS_epi_) has the same linker peptide sequence as that in SaeS [23] but is predicted to have only one transmembrane helix, whose role in the activation of SaeS_epi_ is not known (Fig 1A and S1 Fig). The SaeS_epi_ protein was not detected by the SaeS antibody (Fig 2B), whereas the FLAG-tagged SaeS_epi_ was detected by anti-FLAG-tag antibody (S3 Fig), indicating a significant antigenicity difference between SaeS and SaeS_epi_. More importantly, it did not respond to HNP1 (S_epi_ in Fig 2C). However, when the N-terminal domain of SaeS_epi_ was replaced with that of SaeS, the hybrid SaeS showed a basal kinase activity similar to that of SaeS and responded to HNP1 (S_e-ND_ in Fig 2C), confirming that the N-terminal domain is sufficient to confer a switch function to SaeS. As expected, since their basal kinase activities were not decreased, the promoter activity of P*hla* was not significantly altered by the hybrid SaeS proteins except for the unstable SaeS_ELP_ mutant (Fig 2D).

### The Linker Peptide Restrains the Basal Kinase Activity

**Fig 3:**
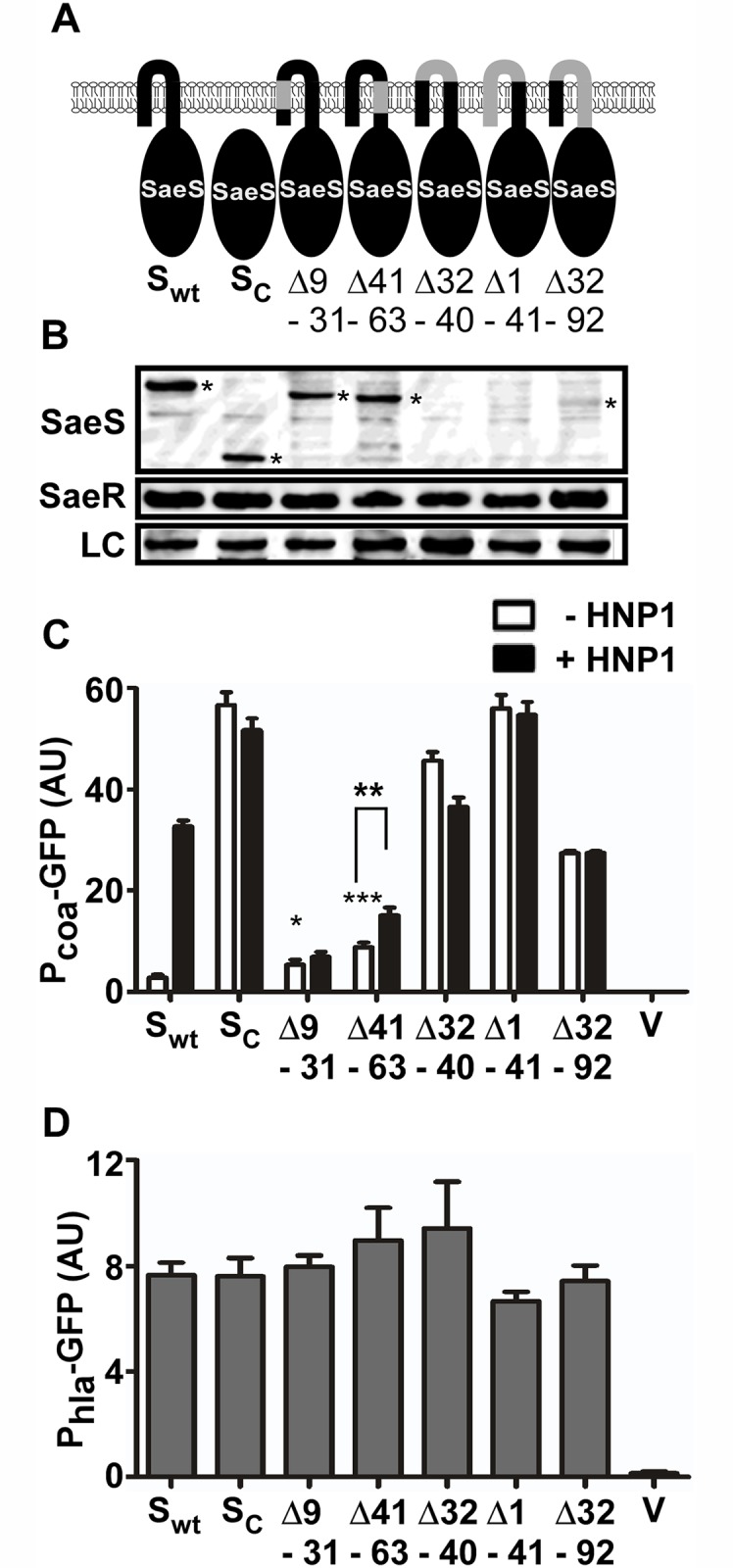
The extracytoplasmic linker peptide restrains the basal kinase activity. (A) A diagram for the deletion mutants of SaeS. The deleted amino acid sequences are indicated in gray. The numbers represent the amino acid positions. wt, wild type; S_c_, cytoplasmic domain of SaeS; Δ, deletion. (B) Expression of the deletion mutants of SaeS analyzed by Western blot. Cells at exponential growth phase were used. SaeS band is indicated by an asterisk. LC, loading control. (C) The effect of the deletion mutations on the transcription of the low affinity target P*coa*. Experiment conditions are the same as in Fig 2C. Error bars represent the standard error of the mean. Unless indicated otherwise, GFP expression was compared with that of the strain carrying wild type SaeS, and statistical significance was assessed by unpaired, two-tailed student’s t-test. *, p < 0.05; **, p < 0.01; ***, p < 0.001; AU, arbitrary unit; V, vector control. (D) The effect of the deletion mutations on the transcription of the high affinity target P*hla*. Error bars indicate the standard error of the mean.

We further investigated the role of each component of the N-terminal domain by deletion mutagenesis. When the first 92 amino acids, which encompasses the entire transmembrane region (9–63 a.a.), were deleted, the resulting SaeS mutant (SaeSc) was locked in the kinase ON state and did not respond to HNP1 (S_c_ in Fig 3A–3C). When each of the transmembrane helices was deleted, the activity of the reporter promoter P*coa* was increased slightly (Δ9–31 and Δ41–63 in Fig 3A–3C) and showed no (Δ9–31) or poor (Δ41–63) response to HNP1. The poor response to HNP1 was not due to the mislocalization of the proteins because the SaeS mutant proteins were still found in the cell membrane (S4 Fig). When the linker peptide was deleted, despite that no SaeS protein was detected (Δ32–40 in Fig 3A and 3B), P*coa* showed a high promoter activity that did not respond to HNP1 (Δ32–40 in Fig 3C). The other two mutants containing the linker peptide deletion (i.e., Δ1–41 and Δ32–92 in Fig 3A) showed similar results: no or low SaeS protein in Western blot analysis but high basal kinase activity that does not respond to HNP1 (Δ1–41 and Δ32–92 Fig 3A–3C and S4 Fig). No significant change was observed in the P*hla* activity (Fig 3D), suggesting that no SaeS mutant protein has drastically decreased kinase activity. Taken together, these results indicate that both the transmembrane helices and the linker peptide play a key role in responding to HNP1 and that the linker peptide is critical for restraining the basal kinase activity of SaeS.

### Alanine Substitutions in the Linker Peptide Alter the Kinase Activity of SaeS

**Fig 4:**
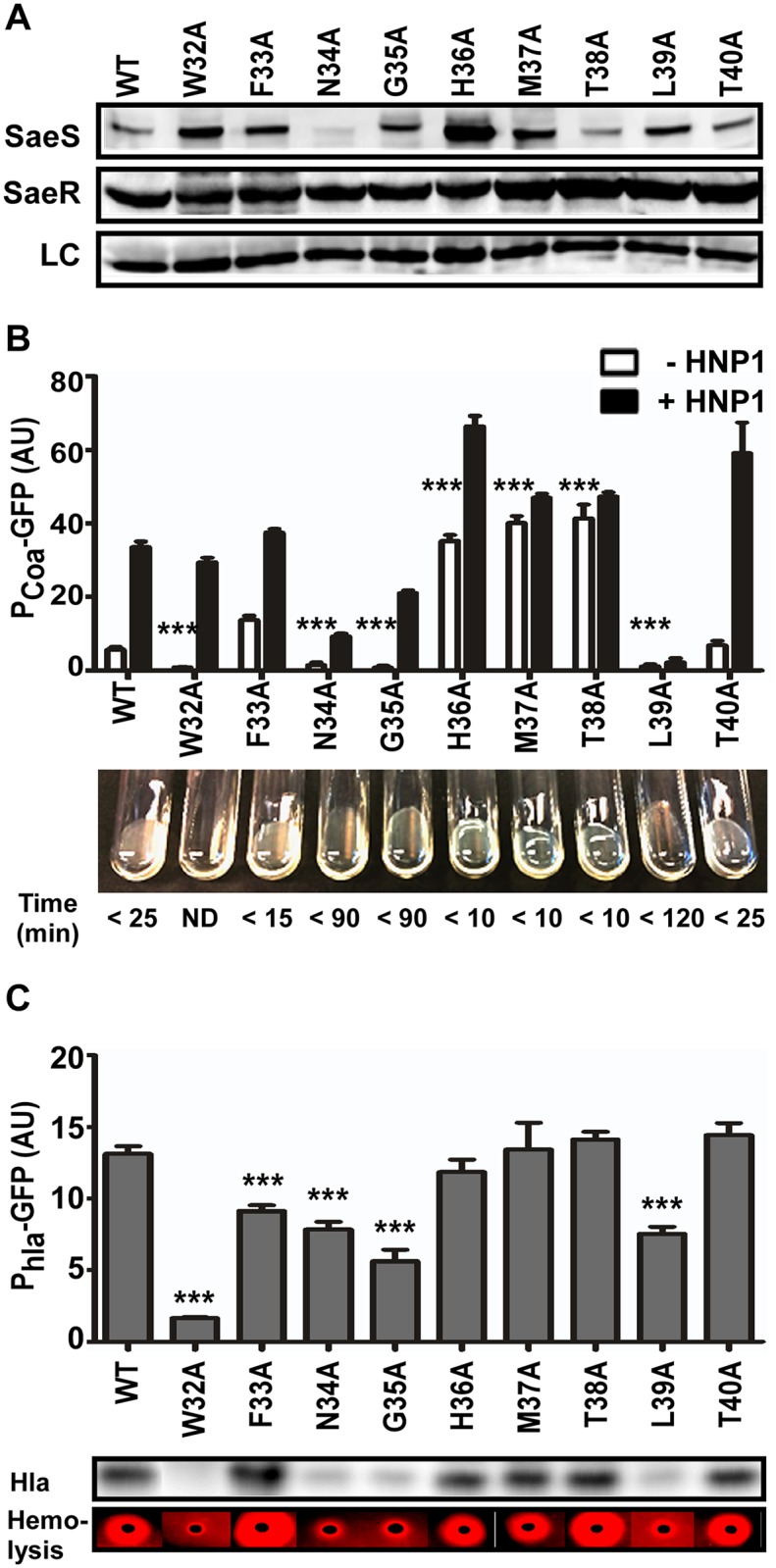
Alanine substitutions in the linker peptide alter the kinase activity of SaeS. (A) Expression of the alanine substitution mutants of SaeS analyzed by Western blot. An equal number of the cells were harvested at exponential growth phase. LC, loading control. (B) The effect of the alanine substitution mutations on the transcription of the low affinity target P*coa* and blood coagulating activity. The promoter activity of P*coa* was measured as described for Fig 2C. Coagulation activity was measured by mixing bacterial culture with rabbit plasma at 37°C. Coagulation time is indicated under each result. Error bars represent the standard error of the mean. WT, wild type; AU, arbitrary unit; ND, not detected. (C) The effect of the alanine substitution mutations on the transcription of the high affinity target P*hla* and the production of alpha-hemolysin (Hla). The production of Hla was examined by Western blot analysis and hemolysis assay on a rabbit blood agar. In the hemolysis assay, red zone indicates complete hemolysis. In the bar graphs, the error bars indicate the standard error of the mean. GFP expressions were compared with that of the strain carrying wild type SaeS, and statistical significance was assessed by unpaired, two-tailed student’s t-test. ***, p < 0.001.

Since the linker peptide appears critical for the switch function of SaeS (i.e., maintaining low basal kinase activity and responding to HNP1), we further examined the role of each amino acid in the linker peptide by alanine scanning analysis. Seven mutant SaeS proteins showed a wild type level of protein expression; however, the N34A and H36A mutants showed either significantly lower (40% of wild type level) or higher expression (3.3 times wild type level), respectively (Fig 4A). In the reporter and coagulase assays, both the uninduced P*coa* transcription and coagulation of rabbit plasma were significantly decreased in the strains carrying W32A, N34A, G35A, or L39A mutant of SaeS, whereas they were greatly increased in those carrying H36A, M37A, or T38A mutant of SaeS (Fig 4B), demonstrating that the basal kinase activity of SaeS can be altered by amino acid changes in the linker peptide. In addition, the P*coa* transcription did not respond to HNP1 in the strains carrying M37A, T38A, or L39A mutant of SaeS, suggesting a critical role of those amino acid residues in responding to the antimicrobial peptide. The P*hla* promoter activity further confirmed the drastic decrease of basal kinase activity in the W32A, N34A, G35A, and L39A mutants of SaeS (Fig 4C). Both Western blot analysis for Hla and the hemolysis assay on blood agar plates correlated with the P*hla* promoter activity (Fig 4C). Intriguingly, by an unknown reason, as compared with wild type strain, the F33A mutant strain showed a lower P*hla* promoter activity despite the fact that P*coa* promoter activity and Hla expression level were rather higher.

### Alanine Substitutions in the Linker Peptide Alter the Autokinase and Phosphotransferase Activities of SaeS

**Fig 5:**
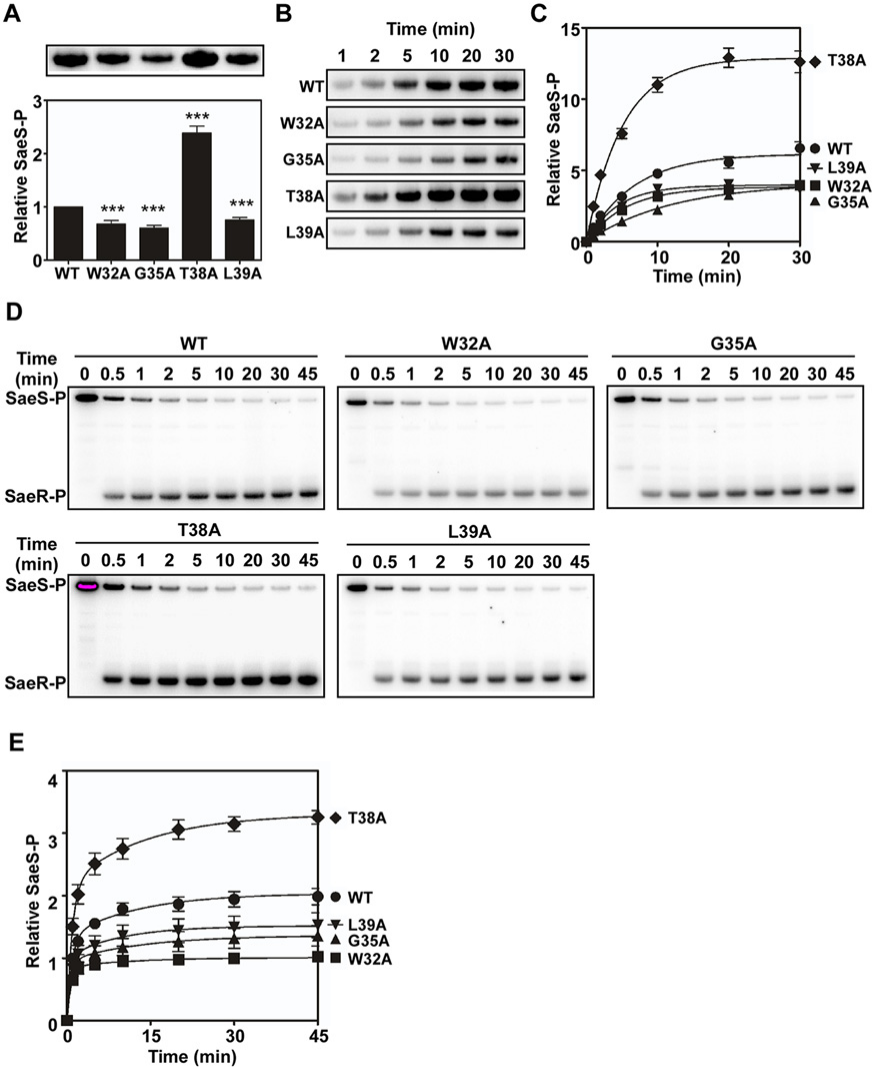
Alanine substitutions in the linker peptide alter the kinase and phosphotransferase activities of SaeS. (A) The autokinase activity of wild type (WT) and select linker peptide mutants of SaeS. The purified MBP-SaeS proteins (5 μM) were incubated with [γ-^32^P] ATP at RT for 20 min. The autoradiograph of the phosphorylated MBP-SaeS (upper panel) is shown with its quantification results in a bar graph (lower panel). (B) Assessment of the autokinase activity of wild type (WT) and select linker peptide mutants for 30 min. The wild-type or the linker peptide mutant MBP-SaeS proteins (5 μM) were mixed with [γ-^32^P] ATP and, at the indicated times, the level of phosphorylated MBP-SaeS was analyzed by phosphor imager analysis. (C) Quantitation of the autophosphorylation assays shown in (B). The plot depicts the levels of MBP-SaeS-P relative to the wild-type MBP-SaeS-P at time 1 min as a function of time. (D) Phosphotransferase activity of the wild type (WT) and select linker peptide mutants of SaeS. Phosphorylated MBP-SaeS (5 μM) was mixed with SaeR (10 μM). At the times indicated, the reaction was stopped and the phosphorylated proteins were analyzed by SDS-PAGE and phosphor imager analysis. (E) Quantification of the phosphotransfer assays shown in (D). Each datum on the plot depicts the level of SaeR-P relative to that of the wild-type SaeS at the initial time (1 min). All data correspond to the mean values of three independent experiments, and error bars show standard deviation. For statistical analyses, unpaired two-tailed student’s t-test was used. ***, p < 0.001

**Fig 6:**
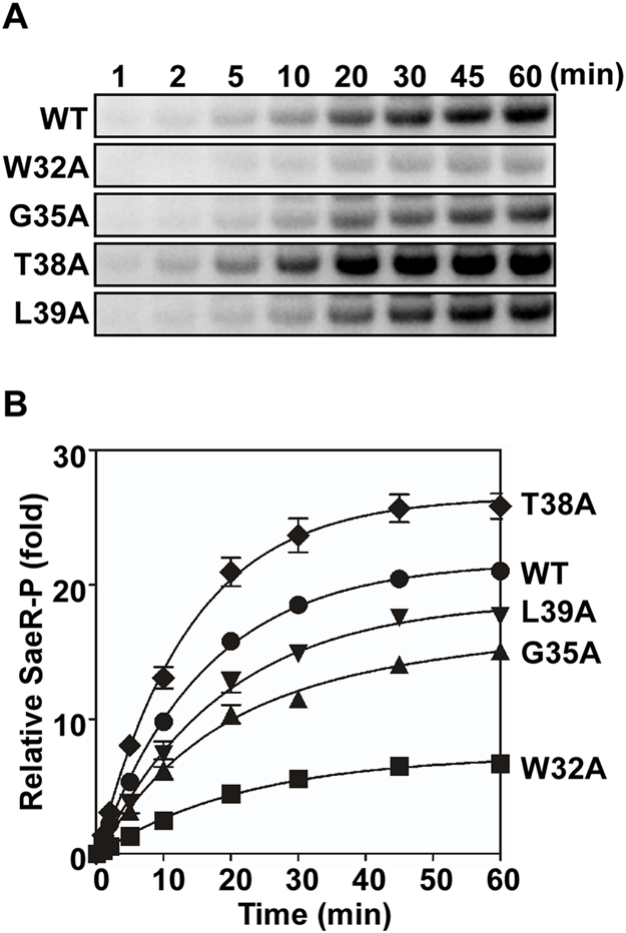
Membrane vesicles harboring the linker peptide mutant SaeS exhibit altered phosphotransferase activity. (A) Levels of SaeR-P following incubation of SaeR, [γ-^32^P] ATP, and membrane vesicles (300 μg) harboring the wild-type or the various linker mutant SaeS proteins. (B) Quantitation of the phosphotransfer assay shown in (A). Each datum of the plot depicts the level of SaeR-P relative to that by the wild-type SaeS at the initial time (1 min). Data correspond to the mean values of three independent experiments, and error bars show standard deviation.

The sensor kinase SaeS has three enzymatic activities: autokinase, phosphotransferase, and phosphatase. To examine which enzyme activity is affected by the alanine substitutions, we compared those enzymatic activities between the wild type and the following mutant SaeS proteins: SaeS W32A and SaeS G35A (decreased basal activity and normal induction by HNP1), SaeS T38A (increased basal activity and no induction by HNP1), and SaeS L39A (decreased basal activity and no induction by HNP1). To compare the autokinase activity, we mixed an equal amount of maltose binding protein (MBP)-SaeS fusion proteins with [γ-^32^P] ATP and compared the levels of phosphorylated SaeS (SaeS-P) at 20 min. As shown, a lower level of phosphorylation was observed with the SaeS mutants that showed lower basal kinase activities in the reporter gene assays (W32A, G35A, and L39A in Fig 5A). Similarly, SaeS T38A, which showed a higher basal kinase activity in the reporter assay (Fig 4B) exhibited 2.5-fold higher autokinase activity, as compared with the wild type MBP-SaeS fusion protein (T38A in Fig 5A). When the autokinase activity was measured in a time-dependent manner, the SaeS T38A protein autophosphorylated two times faster than did the wild-type SaeS protein, whereas the three mutants with lower autokinase activities (i.e., W32A, G35A, and L39A) displayed a slower rate of autophosphorylation (Fig 5B and 5C). Next, to compare the phosphotransferase activity, we autophosphorylated the MBP-SaeS proteins with [γ-^32^P] ATP; then, after eliminating the free nucleotide, the phosphoryl transfer was initiated by adding SaeR. As compared with wild type SaeS, the W32A, G35A, and L39A mutants of SaeS exhibited a slower rate of phosphoryl transfer, whereas the SaeS T38A showed a higher phosphoryl transfer rate (Fig 6D and 6E). Taken together, these results indicate that the mutations in the linker peptide directly alter the autokinase and phosphotransferase activities of SaeS.

The enzymatic assay results from the purified proteins correlated well with the results of the *in vivo* reporter assay in Fig 4B. However, since we used purified MBP-SaeS fusion proteins, it was desirable to confirm the results in conditions more closely resembling native conditions. Therefore, we isolated membrane vesicles from *S*. *aureus* strains harboring either the wild-type SaeS or the linker mutant SaeS and used the membrane vesicles as the source of SaeS in a phosphotransferase assay. Since the protein expression was different among the SaeS proteins (Fig 4A), the SaeS concentration was normalized by Western blot analysis, and an equal amount of SaeS in the membrane vesicles was mixed with [γ-^32^P] ATP and SaeR; then the phosphorylation of SaeR was measured in a time-dependent manner. As compared with the membrane vesicle containing the wild type SaeS protein, the vesicles containing W32A, G35A, and L39A mutants of SaeS showed lower phosphotransferase activities whereas the vesicles containing SaeS T38A mutant displayed a higher phosphotransferase activity (Fig 6), agreeing with the reporter assays (Fig 4B) and the phosphotransferase assays with the MBP-SaeS proteins (Fig 5). These results demonstrate that the linker peptide controls the activity of the SaeRS TCS by altering autokinase and phosphotransferase activity of SaeS.

### Glycine is the Optimal Amino Acid at the Position 35 to Confer the Switch Function to SaeS

**Fig 7:**
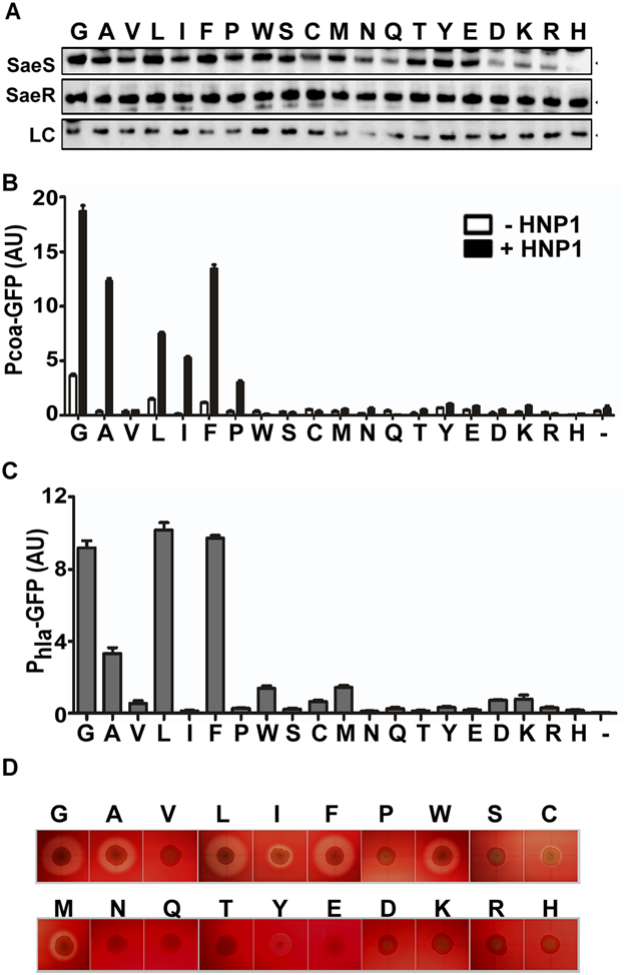
At the position 35, Gly is the optimal amino acid for SaeS switch function. (A) Expression of the substitution mutants of SaeS analyzed by Western blot. Amino acid at position 35 is shown at the top of the panel. An equal number of the cells were harvested at exponential growth phase and used for the analysis. LC, loading control. (B) GFP transcription from the low affinity target P*coa* in strains carrying wild type or mutants of SaeS carrying various amino acid substitutions at the position 35. Experiment conditions are the same as in Fig 2C. HNP1 (5 μg/ml) was used to induce the SaeRS TCS. Error bars represent the standard error of the mean.-, vector control; AU, arbitrary unit. (C) GFP transcription from the high affinity target P*hla*. The error bars indicate the standard error of the mean.-, vector control. (D) The effect of the substitution mutations on the hemolysis activity of *S*. *aureus*. The test strains were grown on a rabbit blood agar overnight.

To investigate the precise role of the amino acids in the linker peptide, we replaced the Gly35 with other 19 amino acids and examined their effects on the switch function of SaeS. As can be seen in Fig 7B, substitutions with any polar amino acids abolished both the basal kinase activity and the response to HNP1. Only G35L and G35F mutants maintained basal kinase activity near wild type level (L and F in Fig 7B–7D). Upon induction by HNP1, five substitution mutants (A, L, I, F, and P) showed a varying degree of responses; however, none of the mutants reached the response level of the wild type (G), suggesting that glycine is the optimal amino acid for SaeS switch function at the position 35.

Results from the Gly35 substitution experiment indicate that the kinase activity of SaeS varies depending on the occupying amino acids. To examine this residue-dependent effect further, we generated a few additional substitutions for other positions and examined their effect on the SaeS switch function. As shown in S5 Fig, SaeS mutant proteins showed disparate basal kinase activities and/or HNP responses depending on the occupying amino acids. Although SaeS F33V showed higher basal kinase activity, the SaeS F33Y mutant showed lower basal kinase activity (F33 in S5B and S5C Fig). The N34Q mutant showed drastically reduced basal kinase activity, whereas N34L showed a wild type level of basal kinase activity (N34 in S5B and S5C Fig). As compared with SaeS L39A, which lost both basal kinase activity and response to HNP1 (Fig 4B), SaeS L39V showed significantly decreased basal kinase activity but normal response to HNP1 (L39V in S5 Fig). These results further confirm that the kinase activity of SaeS sensitively responds to the amino acid changes in the linker peptide.

### The Wild Type SaeS Shows the Most Robust Switch Function in Clinically Relevant Environments

**Fig 8:**
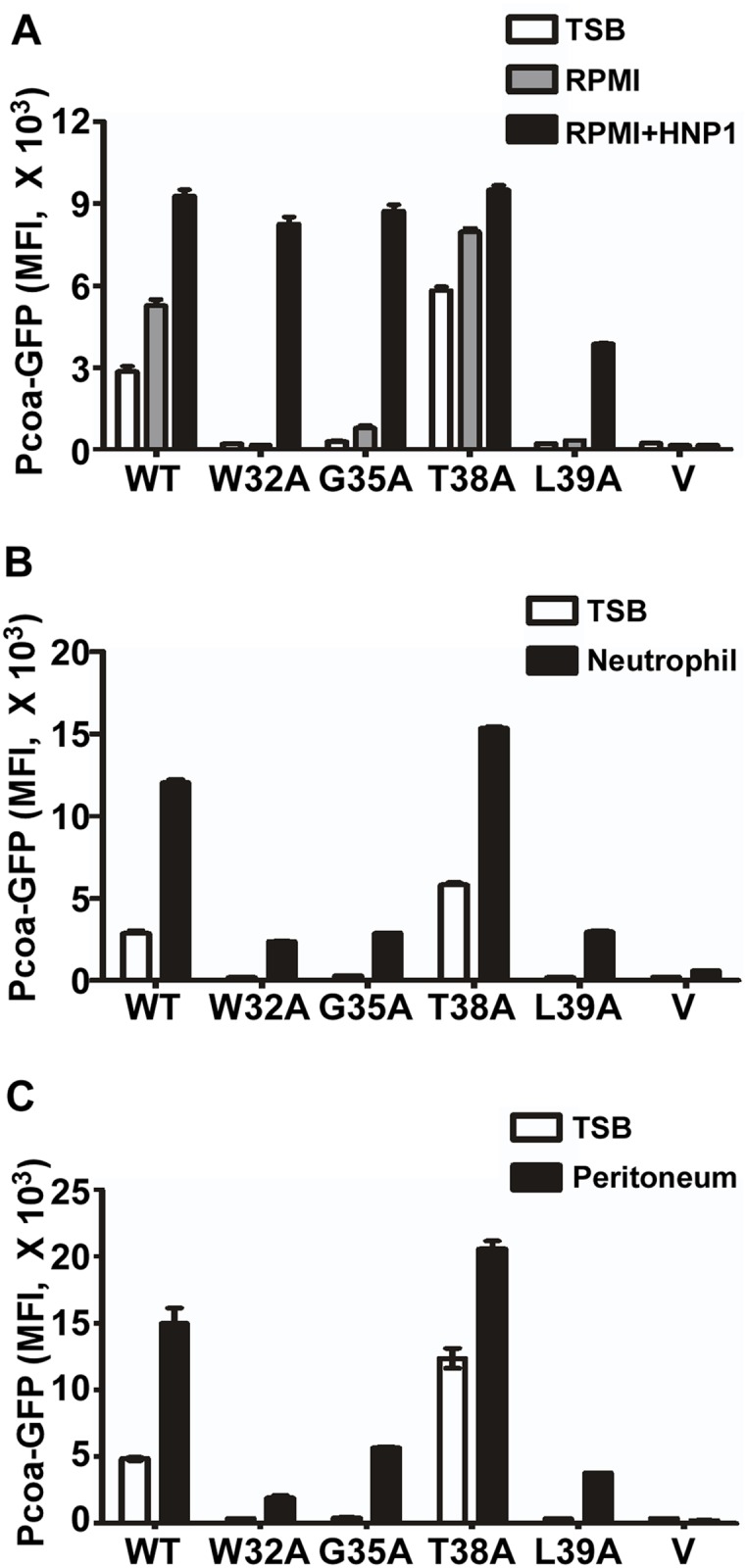
The wild type SaeS shows the most robust switch function in clinically relevant environments. (A) The effect of the growth medium RPMI on the switch function of wild type and select linker peptide mutants of SaeS. Cells were grown in RPMI until exponential growth phase. For induction of the SaeRS TCS, HNP1 (5 μg/ml) was added, and the culture was further incubated for 2 h. Expression of GFP was measured by flow cytometry. The GFP expression in TSB is also shown for comparison. Error bars represent the standard error of the mean. WT, wild type; V, vector control; MFI, mean fluorescence intensity. (B) The effect of the linker peptide mutations on the transcription of the low affinity target *Pcoa* in the presence of human neutrophils. Human neutrophils were purified and mixed with the test strains in the ratio of 1:10. Two hours later, the GFP expression of the strains was measured by flow cytometry analysis. (C) The effect of the linker peptide mutations on the *sae* activity during murine peritoneum infection. The test strains at exponential growth phase were injected into murine peritoneum. Two hours later, the peritoneal exudates were obtained, and GFP expression of the strains was measured by flow cytometry analysis.

Next, we compared the switch function of the wild type and select mutants of SaeS in various environmental conditions. We subjected the test strains to TSB, RPMI (Roswell Park Memorial Institute medium), human neutrophil, and murine peritoneum; then we measured the P*coa* activity by flow cytometry analysis as an indicator for the SaeS kinase activity. As compared with TSB, in RPMI, the wild type and SaeS T38A mutant showed heightened basal kinase activities, which responded to HNP1 (Fig 8A). On the other hand, the W32A, G35A and L39A mutant SaeS proteins showed almost no basal kinase activity; however, in RPMI, the W32A and G35A mutant SaeS showed a wild type level of induction in response to HNP1, and even the SaeS L39A, which did not respond to HNP1 in TSB (Fig 4), did respond to HNP1 in RPMI (Fig 8A). These results suggest that the switch function of SaeS can be altered by growth conditions.

When the test strains were subjected to more clinically relevant conditions such as neutrophil and murine peritoneal infection model, the W32A, G35A, and L39A mutant proteins showed significantly lower kinase activity as compared with the wild type and the T38A mutant SaeS (Fig 8B and 8C). Considering the elevated basal kinase activity of the T38A mutant, overall, the wild type SaeS showed the most robust and stable switch function in those environmental conditions, indicating that the amino acid sequence of the linker peptide was highly optimized in SaeS. It should be noted that mice do not produce HNP-like antimicrobial peptides [24]. Therefore, the induction of SaeS kinase activity in murine peritoneum suggests the existence of a novel *sae*-inducing signal in mice [25].

### The Kinase Activity of SaeS Correlates with the Virulence of *S*. *aureus* in a Murine Infection Model

**Fig 9:**
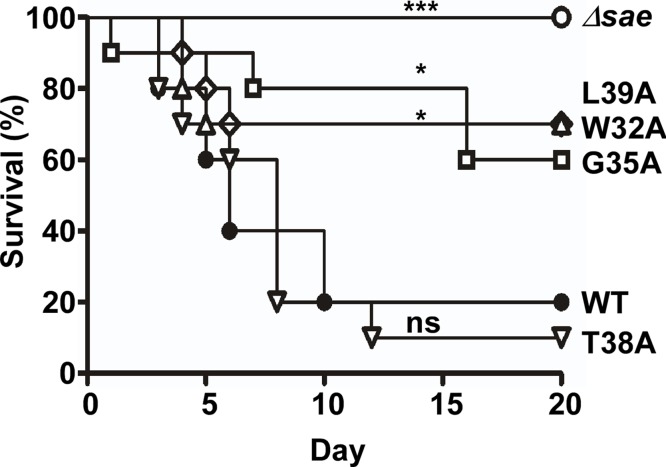
The linker peptide mutants exhibiting lower kinase activity show attenuated virulence in a murine model of infection. The test strains were grown in TSB until exponential growth phase; then the test strains (10^7^ cfu) were administered into Balb/c mice via retro-orbital route. The infected mice were observed for 20 days. The differences in the survival were analyzed by Log-rank (Mantel-Cox) test. *, p < 0.05; ***, p < 0.001; ns, not significant. The experiment was repeated, and similar results were observed.

Finally, we assessed the effect of the linker peptide-mediated alterations of the SaeS kinase activity on *S*. *aureus* virulence in a murine model of infection. The strain carrying SaeS T38A, which has elevated basal kinase activity, showed wild type level of virulence while the strains carrying SaeS with lower basal kinase activity (i.e., SaeS W32A, G35A, and L39A) were attenuated (Fig 9). In parallel, the strain harboring the deletion of the *sae* operon lost its virulence in infected mice (Fig 9). These results further confirm the physiological relevance of the linker peptide-mediated control of the SaeS’s kinase activity in the bacterial pathogenesis.

## Discussion

Unlike typical sensor histidine kinases, SaeS lacks a ligand binding domain, and its N-terminal domain is comprised of two transmembrane helices and a linker peptide of nine amino acids. Due to its essential role in staphylococcal virulence and bacterial survival in the host, the sensing mechanism of SaeS has been a focus of extensive research. However, the role of the N-terminal domain, especially the linker peptide, has not been fully understood. In this study, we established that a single amino acid change in the linker peptide can alter the kinase activity of SaeS and cause differential expression of SaeR-regulated genes, resulting in altered virulence of the important human pathogen. In addition, our results suggest that the amino acid sequence of the linker peptide is highly optimized for the proper control of the kinase activity of SaeS.

Recently, Flack et al has reported that the three amino acid residues (i.e., M31, W32, and F33), not the entire linker peptide, are critical for the kinase activity of SaeS [23]. This conclusion is based on the observation that the production of alpha-hemolysin (Hla) was greatly reduced in *S*. *aureus* strains carrying SaeS with an M31A, W32A, or F33A substitution. However, since the activity of P*hla* is sensitive only to a large decrease in the kinase activity and insensitive to a moderate decrease or increase of SaeS’s kinase activity [18] (Figs 2–4, and S2 Fig), the Hla production assay alone cannot detect all changes in the kinase activity of SaeS (e.g., the increase of the kinase activity by H36A, M37A, and T38A substitutions.) In addition, since the authors made only alanine substitutions, the amino acid-dependent changes in the kinase activity of SaeS could not be detected. For example, based on the observation with SaeS M31A, the authors suggested M31 is essential for the kinase activity of SaeS; however, when M31 was substituted with cysteine, the resulting mutant showed constitutively elevated kinase activity (S5D and S5E Fig), demonstrating that M31 itself is not critical for the kinase activity. Intriguingly, sequence alignment of SaeS homologs from Firmicutes revealed that M31 and L41 are highly conserved (S6A–S6E Fig). In addition, sequence alignment of 330 GraS homologs showed the conservation of D35 and Y45 (S6F Fig). We speculate that those conserved amino acid residues play a critical role in hitherto unknown functions such as protein-protein interactions, protein-lipid interactions at the interface, or protein folding. Moreover, the sequence analysis also showed that each bacterial family uses a different set of amino acids for the linker peptide (S6B–S6E Fig), implying that the amino acid sequences of the linker peptide might be differentially optimized for the particular external signals sensed by each bacterial family.

The linker peptide is likely to contribute to the switch function of SaeS in two ways. First, it restrains the basal kinase activity of SaeS so that SaeS can function as a molecular switch from the partially ON state to the fully ON state. Without the linker peptide, the kinase activity of the resulting SaeS variant is constantly elevated, even higher than HNP1-induced level, and does not respond to HNP1 (Δ32–40 in Fig 3B and 3C). Second, when *S*. *aureus* experiences the host signals, the linker peptide is expected to transduce the external signal input to control the kinase activity of SaeS, possibly via conformational changes. The kinase activity of SaeS sensitively responds to alanine substitutions in the linker peptide (Fig 4). Since amino acid substitutions with different amino acids resulted in distinct kinase activities (Fig 7 and S5 Fig), it is more likely that the conformational changes caused by the amino acid substitutions, not the biochemical traits of the substituting amino acid itself, altered the enzyme activities of SaeS (Figs 5 and 6). Therefore, we presume that, in the presence of HNP1, the linker peptide undergoes conformational changes, and the conformational changes alter the kinase activity of SaeS, possibly via the HAMP (Histidine kinase, Adenyl cyclase, Methyl-accepting proteins, Phosphatase) domain (amino acid 61–114).

In this study, we showed that the kinase activity of SaeS can be modulated by amino acid changes in the linker peptide. However, our study does not answer the question of how HNP1 activates the SaeRS TCS. One possibility is that HNP1 activates SaeS by directly binding to the N-terminal domain of SaeS, possibly through interaction with the linker peptide. However, it has been shown that, in certain strains of *S*. *aureus* (e.g., ISP479R and COL), the SaeRS TCS does not respond to HNP1 despite the fact that these strains possesses the wild type SaeS protein [26], indicating that SaeS alone is not sufficient to respond to HNP1. In addition, our multiple attempts including co-immunoprecipitation failed to observe any direct interaction between HNP1 and SaeS. Therefore, it is more likely that HNP1 activates SaeS indirectly via a hitherto unidentified receptor molecule(s), and the N-terminal domain of SaeS receives the signal from the receptor molecule as a signal transfer region [9]. Indeed, the BceS/LiaS-like IM-HKs alone cannot perceive stimuli and require additional components such as ABC transporter (BceS-like IM-HKs) and membrane protein (LiaS-like IM-HKs) for signal sensing [7,8,27,28,29,30]. Recently, Omae et al showed that the apolipophorin of silkworms represses the kinase activity of SaeS via binding to lipoteichoic acid [21], indicating that the kinase activity of SaeS can be modulated by interaction with other molecules in the membrane. Although the SaeRS TCS has two auxiliary proteins, SaeP and SaeQ, located in the membrane, they are dispensable for the HPN1 sensing [17]. Therefore, the HNP1-sensing is likely carried out by a receptor molecule(s) in the membrane. Upon binding to HNP1, the HNP1 receptor is expected to induce a conformational change in the N-terminal domain similar to those elicited by H36A, M37A or T38A (Fig 4B) by direct protein-protein interaction.

Recently T. Mascher postulated that the N-terminal region of IM-HKs is not a signal sensor but a signal transfer region, and that it transduces the external signals to the kinase domain via direct protein-protein interaction with the true sensor molecules [9]. Our results provide indirect evidence that, if such a true sensor exists, the interaction of the N-terminal domain with true sensor molecule(s) can modulate the kinase activity of the sensor kinase. We propose that, in the signal transfer process, the entire N-terminal domain of SaeS (i.e., two transmembrane helices and the linker peptide) works as a coherent unit in a manner of a tripwire. In this “tripwire” model, the overall conformation of the entire N-terminal domain is the key determinant in controlling the kinase activity of the sensor kinase. Any stimulus that elicits conformational changes in the N-terminal domain is expected to affect the kinase activity of the sensor kinase either as a repressor or an activator, depending on the nature of the conformational change. In the case of SaeS, if the resulting conformation is similar to that elicited by a W32A, G35A, or L39A substitution, the stimulus will be a repressor while, if the conformation is similar to those elicited by H36A, M37A, or T38A, the stimulus will act as an activator. This tripwire model can also explain how the kinase activity of SaeS is modulated by structurally unrelated molecules such as HNPs, apolipophorin, and beta-lactam antibiotics [21,26,31]. The receptors for those SaeS modulators are expected to be distinct molecules, possibly interacting with different residues of the N-terminal domain of SaeS. Therefore, it is possible that, by separating the sensing receptor from the signal transfer region, IM-HKs with simple N-terminal domain are able to respond to diverse external signals without losing specificity.

## Materials and Methods

### Ethics Statement

The human subject experiment (i.e., purification of human neutrophils) was approved by the Indiana University Institutional Review Board (Study number: 1010002390). Before taking blood, informed written consent was obtained from each human subject. The animal experiment was performed by following the Guide for the Care and Use of Laboratory Animals of the National Institutes of Health. The animal protocol was approved by the Committee on the Ethics of Animal Experiments of the Indiana University School of Medicine-Northwest (Protocol Number: NW-34). Every effort was made to minimize suffering of the animals.

### Bacterial Strains, Plasmids and Culture Conditions

The bacterial strains and plasmids used in this study are listed in S1 Table. *Escherichia coli* was grown in Luria-Bertani broth (LB), while *S*. *aureus* was cultured in tryptic soy broth (TSB) or Roswell Park Memorial Institute medium (RPMI). For transduction of plasmids, heart infusion broth (HIB) supplemented with 5 mM CaCl_2_ was used. When necessary, antibiotics were added to the growth media at the following concentrations: ampicillin, 100 μg/ml; kanamycin, 30 μg/ml; erythromycin, 10 μg/ml; and chloramphenicol, 5 μg/ml.

### DNA Manipulation

Unless stated otherwise, all restriction enzymes and DNA modification enzymes were purchased from New England Biolabs. For PCR amplification, the Phusion DNA polymerase (New England Biolabs) was used. Plasmids and genomic DNA were extracted with Zippy^TM^ plasmid miniprep kit (Zymo Research) and GenElute^TM^ Bacterial Genomic DNA kit (Sigma), respectively, according to the manufacturer’s instruction. Plasmid DNA was introduced into *E*. *coli* by the method of Hanahan and Meselson [32] and into *S*. *aureus* RN4220 by electroporation with a gene pulser (Bio-Rad). Subsequent transduction of the plasmids into target strains of *S*. *aureus* was carried out with ϕ85.

### Construction of NMΔ*phoB* Strain and PhoB Fusions

To construct NMΔ*phoB* strain, DNA sequences 1 kb upstream and downstream of *phoB* were PCR-amplified using the primer pairs P2002/2003 and P2004/2005 (S2 Table). The target vector pIMAY was also PCR-amplified with primer pairs P1986/1987 (S2 Table). The PCR products were assembled by the ligation independent cloning method [33]. First, the insert DNA and vector PCR products were treated with T4 DNA polymerase for 30 min at room temperature. Then the DNA fragments were mixed and incubated at 37°C for 30 min and transformed into *E*. *coli*. The pIMAY containing the *phoB* deletion cassette was isolated and electroporated into RN4220. Subsequently, the plasmid was moved into Newman strain by ϕ85-mediated transduction. The *phoB*-deletion was carried out by following the procedures previously reported [34] and verified by PCR amplification of the gene locus.

To generate PhoB fusions at Arg6 (R6), Gly35 (G35) and Asn71 (N71) of SaeS, *phoB* fragments lacking a signal peptide sequence were PCR-amplified with the following primer pairs: P2164/2165 for R6-PhoB, P2107/2108 for G35-PhoB, and P2168/2169 for N71-PhoB (S2 Table). The target vector pCL55-*saeRS* was PCR-amplified with primer pairs P2162/2163 (R6-*phoB*), P2103/2104 (G35-*phoB*), and P2166/2167 (N71-*phoB*), respectively (S2 Table). All resulting PCR products were treated with T4 DNA polymerase for 30 min at room temperature. The insert *phoB* fragment and its corresponding vector DNA were mixed and incubated at 37°C for 30 min; then the mixture was transformed first into *E*. *coli*, and subsequently into RN4220 and its target strain, NMΔ*phoB*. The test strains were inoculated onto tryptic soy agar plate containing XP (5-bromo-4-chloro-3-indolyl phosphate, toluidine salt, 100 μg/ml, Sigma) [35].

### Construction of Plasmids

To generate the cytoplasmic domain of SaeS (SaeSc in Fig 3), the linker peptide deletion mutant of SaeS (Δ32–40 in Fig 3) and the hybrid SaeS with the linker peptide from GraS (S_ELP_ in Fig 2), DNA fragments were amplified from pCL55-*saeRS* with the phosphorylated primer pairs P1887/1888, P1958/1960 and P1961/P1962 (S2 Table). The amplified fragments were circularized with T4 ligase and then transformed into *E*.*coli*.

To generate SaeS_ND_, SaeS_epi_ and SaeS_e-ND_ in Fig 2, a sequence- and ligation- independent cloning (SLIC) method was used [36]. The insert DNA fragments were PCR-amplified with the primer pairs P2061/2062, P2143/2144, P2145/2146, respectively, using genomic DNA of USA300 or *S*. *epidermidis* RP62a as a template (S2 Table). On the other hand, the target vectors were PCR-amplified with the following primer pairs: P2059/2060 for SaeS_ND_, P2141/2142 for SaeS_epi_, and P2147/2142 for SaeS_e-ND_ using pCL55-*saeRS* as a template. All the resulting PCR products were treated with T4 DNA polymerase for 30 min at room temperature. Then the PCR products and their vector counterparts were mixed, incubated at 37°C for 30 min, and the mixture was transformed into *E*. *coli*.

To generate SaeS_TM_ (S_TM_ in Fig 2), the phosphorylated primers P2226 and P2227 (S2 Table) were used to amply DNA fragment from pCL55-*saeRS*_*ND*_ (S_ND_ in Fig 2A). The amplified fragments were circularized with T4 ligase and then transformed into *E*.*coli* directly.

Alanine scanning of the extracytoplasmic linker peptide and mutagenesis of Gly35 of the linker peptide were carried out as described by Ho et al[37]. The presence of the mutation was verified by DNA sequencing.

To generate a parent plasmid for the promoter-*gfp* (green fluorescence protein) fusions, the *gfp* fragment was PCR-amplified with primer pair P1969/1970 using pSW4-GFP as a template [38]. The PCR product was digested with *Kpn*I and *Xho*I and inserted into pYJ335, resulting pYJ-*gfp*. To generate *gfp* fusions for the coagulase promoter (P*coa*) and alpha hemolysin promoter (P*hla*), we used a ligation independent cloning method [33]. First, vector DNA was PCR-amplified from pYJ-*gfp* using the primers P1969 and P1747 (S2 Table); then the insert DNA fragment containing the promoter sequence was amplified with primer pairs P1973/1974 for P*coa*, and P1992/1993 for P*hla* (S2 Table). The PCR products were treated with T4 DNA polymerase in the presence of dCTP (vector) or dGTP (insert DNA) and mixed together. The DNA mixture was used to transform *E*. *coli* DH5α. Once verified, all plasmids were electroporated into *S*. *aureus* strain RN4220 and subsequently transduced into the *sae*-deletion mutant of *S*. *aureus* strain Newman (NMΔ*sae*) with ϕ85.

### Construction of FLAG-tagged SaeS proteins

Vector DNA was PCR-amplified from pCL55 with primers P1729/P1859 (S2 Table). For insert DNAs, the *saeRS* region was PCR-amplified from pCL-*saeRS* with P167/P365 (for SaeS-FLAG), pCL-*saeRS*_*ELP*_ with P167/P365 (for SaeS_ELP_-FLAG) or pCL-*saeRSepi* with P167/P366 (for SaeS_epi-_FLAG). The PCR products were treated with T4 DNA polymerase, mixed together, and transformed into *E*.*coli*.

### Fractionation of Cell Components

Overnight cultures of *S*. *aureus* strains were diluted 1:100 in fresh TSB and grown at 37°C for 6 h. Cells were collected by centrifugation, suspended in TSM (50 mM Tris HCl, 0.5 M sucrose, 10 mM MgCl_2_, pH 8.0) containing lysostaphin (40 μg/ml), and incubated at 37°C for 30 min. After centrifugation (4,600 ×g, 5 min), the protoplast in the pellet was suspended in membrane buffer (100 mM Tris HCl, 100 mM NaCl, 10 mM MgCl_2_, pH 8.0) and subjected to sonication. Membrane fractions were recovered by ultracentrifugation (120,000 ×g) at 4°C for 30 min and suspended in 1× TKMG buffer (50 mM Tris-HCl, 50 mM KCl, 1 mM MgCl_2_, 25% glycerol, pH 8.0). The supernatant was designated cytoplasmic fraction. All samples were subjected to SDS-PAGE, followed by Western blot analysis.

### Protein Purification

The MBP-SaeS-His_6_ and SaeR-His_6_ proteins were overproduced in *E*.*coli* BL21 (DE3) harboring plasmids pMCSG19-*saeS* or pET28a-*saeR*. Overnight cultures were inoculated into fresh LB broth, and the proteins were expressed by the addition of 1 mM of isopropyl-1-thio-β-D-galactopyranoside (IPTG) to the fresh culture. The bacterial culture was further incubated at 16°C for 16 h (MBP-SaeS-His_6_) or at 37°C for 6 h (SaeR-His_6_). The proteins were purified with Ni-column chromatography (Qiagen) by following the manufacturer’s recommendations. The purified MBP-SaeS-His_6_ and SaeR-His_6_ were dialyzed first in 1× TKM buffer (50 mM Tris-Cl, 50 mM KCl, 1 mM MgCl_2_, pH 8.0) and 1× TBS buffer (10 mM Tris-HCl, 138 mM NaCl, 2.7 mM KCl, pH 7.5), respectively, and then in TKM or TBS buffer containing 25% glycerol. The purified proteins were concentrated with Amicon Ultracell-30 (MW 30,000; Millipore) for MBP-SaeS-His_6_ or Ultracell-15 (MW 10,000; Millipore) for SaeR-His_6_. Protein concentration was determined by the bicinchoninic acid assay (Bio-Rad), and the purified proteins were stored at -80°C until used.

### Autokinase Assay to Determine the Rate of SaeS Autophosphorylation

The MBP-SaeS-His_6_ (5 μM) protein was incubated with 30 μCi of [γ-^32^P] ATP in 70 μl of TKM buffer. The reaction was started with the addition of ATP to the mixture at room temperature. At various time points, the reaction was stopped by mixing a 7 μl aliquot with 6× SDS sample buffer. The samples were kept on ice until loaded onto a 10% Bis-Tris gel (Invitrogen). After electrophoresis, the gel was autoradiographed, and the degree of phosphorylation was determined with phosphor imaging plate (GE), a Typhoon FLA 7000 imaging system, and Multi Gauge software (Fuji Film). The data were fitted using nonlinear regression to a one-phase exponential association (Prism 5, GraphPad). Data represent mean values of at least three independent experiments.

### Phosphotransferase Assay

To phosphorylate MBP-SaeS-His_6_, 10 μM of MBP-SaeS-His_6_ was mixed with 0.1 mM of ATP containing 30 μCi [γ-^32^P] ATP in TKM buffer and incubated at room temperature for 30 min. Excess [γ-^32^P] ATP was removed with a Micro Bio-Spin 6 Column (Bio-Rad) equilibrated with TKM buffer. Seven microliters of the phosphorylated MBP-SaeS-His_6_ (MBP-SaeS-His_6_-P) were kept as a reference. To start the phosphotransfer reaction, the MBP-SaeS-His_6_-P protein was mixed with 10 μM of SaeR-His_6_ in TKM buffer and incubated at room temperature. The reaction was stopped at various time points by mixing a 7 μl aliquot with 6× SDS sample buffer. Samples were kept on ice until SDS-PAGE. After electrophoresis, the gel was autoradiographed, and the degree of phosphorylation was determined as described above. Data represent mean values of at least three independent experiments.

### Preparation of Membrane Vesicles Harboring the SaeS Protein

*S*. *aureus* strains were grown at 37°C to exponential growth phase (OD_600_ ≈ 0.5) at 37°C. Cells were collected, washed once with 10 mM Tris-HCl (pH 8.0), and suspended in TSM buffer (20 mM Tris-HCl, 0.5 M sucrose, 10 mM MgCl_2_, pH 8.0) containing lysostaphin (40 μg/ml), followed by incubation at 37°C for 30 min. After centrifugation (4,600 ×g, 5 min), the pellet was suspended in ice-cold membrane buffer (10 mM Tris-HCl, 100 mM NaCl, 10 mM MgCl_2_, pH 8.0) and subjected to sonication. Non-ruptured protoplasts were removed by a brief centrifugation at 4,600 ×g, and the membrane fraction was recovered after a 45 min centrifugation at 45,000 ×g (Beckman L8-55). The membranes were suspended in 10 mM Tris-HCl (pH 8.0), 2 M KCl and centrifuged for 30 min at 120,000 ×g. The supernatant was discarded, and the pellet was suspended in 10 mM Tris-HCl (pH 8.0), 5 mM EDTA. Finally, the membranes were suspended in 1× TKMG buffer (50 mM Tris-HCl, 50 mM KCl, 1 mM MgCl_2_, 25% glycerol, pH 8.0). The protein concentration was determined by the bicinchoninic acid assay (Bio-Rad) and immunoblotting with anti-SaeS antibodies. The membranes were stored at -80°C until used.

### Phosphotransferase Assay Using Membrane Vesicles Harboring the SaeS Protein

Three hundred microgram of membrane vesicles harboring either the wild-type SaeS or the linker peptide mutant SaeS proteins and 10 μM of the purified SaeR-His_6_ proteins were incubated with 20 μCi of [γ-^32^P] ATP (3000 Ci/mmol; Perkin Elmer) in TKM buffer at room temperature. A 7 μl aliquot was mixed with 6× SDS sample buffer at different time points to stop the reaction. The phosphorylated SaeR-His_6_ proteins were separated by 10% Bis-Tris SDS-PAGE and determined by quantifying the [^32^P]-labeled species using a Typhoon FLA 7000 imaging system and phosphor imaging plate (Fuji Film) followed by quantification with Multi Gauge software (Fuji Film). The data were fitted using nonlinear regression to a one-phase exponential association (Prism 5, GraphPad). Data correspond to mean values of at least three independent experiments.

### Isolation of Peripheral Blood Neutrophils and Serum Collection

Peripheral blood neutrophils were isolated from healthy adult blood donors by a method of dextran sedimentation and discontinuous Percoll gradient, as previously described [39]. Remaining red blood cells were removed by hypotonic solution (eBioscience), and neutrophil purity was determined by flow cytometry with anti-CD3 (OKT3, eBiosience) and anti-CD16 (B73.1, eBioscience). Purified neutrophils were maintained in RPMI 1640 medium supplemented with 10% human serum. For preparation of the human serum, non-heparinized human blood was allowed to clot at 37°C for 1 h and centrifuged at 12,000 ×*g* for 15 min. Supernatant serum was collected, filtered through 0.22 μm and stored at -80°C.

### GFP Reporter Assays

We performed the GFP reporter assays using either microplate reader or flow cytometry.

#### Microplate reader assay

For the P*coa*-GFP assays, overnight cultures of the test strains were diluted 1:100 into fresh TSB (2 ml) and incubated at 37°C with shaking (200 rpm). After 2 h incubation, each culture was divided into two (1 ml each), and human neutrophil peptide 1 (HNP1, 5 μg/ml) was added to one sample and further incubated at 37°C for 2 h. For the P*hla*-GFP assays, overnight cultures were diluted 1:100 into fresh TSB (2 ml) and incubated at 37°C with shaking (200 rpm). At 4 h post incubation, 100 μl of cell cultures was placed in a black 96-well plate in duplicate, and the fluorescence (485 nm excitation, 538 nm emission) was measured in a Perkin-Elmer Envison 2103 multilabel reader. The fluorescence was normalized by OD_600_. For Western blot analyses, cells were collected from the normalizedcell cultures (OD_600_≈ 2.0) by centrifugation and stored at -80°C.

#### Flow cytometry

To assess the effect of different growth medium, overnight TSB cultures of the test strains containing P*coa*-GFP were diluted 1:100 with fresh TSB or RPMI and grown at 37°C to exponential growth phase (OD_600_ = 0.5); each culture was divided into two, and HNP1was added to one sample as described earlier. At 2 h post incubation, *S*. *aureus* cells were collected by centrifugation, washed with and suspended in PBS. GFP expression was measured by fluorescence intensity with MACSQuant (Miltenyi) in FL-1 channel. Data were analyzed using the FlowJosoftware (Tree Star).

For the P*coa*-GFP assays during human neutrophil infection, 2 × 10^5^ neutrophils were added to a 24-well tissue culture plate and allowed to adhere at 37°C for 1 h. Then *S*. *aureus* cells (2 × 10^6^ cfu) were added to the neutrophils (MOI = 10), and plates were centrifuged at 300 ×*g*, 4°C for 8 min and incubated at 37°C. At 2 h post incubation, 1% saponin (0.1% final, Sigma) was added into each well, and samples were incubated on ice for 15 min. *S*. *aureus* cells were collected by centrifugation at 200 ×*g* and suspended in PBS. GFP expression was measured as described above.

For the P*coa*-GFP assays during murine infection, overnight cultures of the test strains were diluted 1:100 in fresh TSB and further incubated at 37°C. At 2 h post incubation, *S*. *aureus* cells were collected by centrifugation, washed with and suspended in sterile PBS (1 × 10^9^ cfu ml^-1^). The bacterial suspension (2 × 10^8^ cfu in 200 μl) was administered into sex-matched eight week-old Balb/c mice via intraperitoneal injection. Two hours later, peritoneal lavage was carried out with 2 ml PBS and a 3 ml syringe with an 18 gauge needle, and the lavage fluid was centrifuged at 3000 ×g to collect both bacterial and murine cells. To lyse murine cells, the collected cell mixture was treated with cold sterile water (pH 10.5) at room temperature for 15 min; then the murine cell debris and remaining murine cells were discarded by centrifugation at 200 ×*g*. The collected *S*. *aureus* cells were suspended in PBS, and GFP expression was measured as described above.

Every GFP assay was independently repeated at least three times, and similar results were observed each time.

### Western Blot Analysis

SaeS, SaeR, and a loading control were detected from whole cell lysates harvested during the fluorescence P*hla* or P*coa* reporter assay. Cell pellets were suspended in 20 mM Tris-HCl buffer (pH 8.0) containing a protease inhibitor cocktail (Complete mini, Roche), and cells were lysed using lysostaphin (40 μg/ml) and DNaseI in a 37°C heat block for 30 min. An equal volume of 2× SDS loading buffer was added to the cell lysates, followed by heating for 5 min. After brief centrifugation, 6 μl of each sample was subjected to 12% SDS-PAGE, and proteins were transferred to Protran BA nitrocellulose membranes (Whatman). Membranes were blocked with 10% skim milk (wt/vol) in TBST (20 mM Tris-HCl, 137 mM NaCl, and 0.05% Tween 20, pH 7.6) for 1 h. SaeR or SaeS antibody was diluted 1:2000 in TBST containing BSA (1 mg/ml) (TBST-B) and incubated with the membranes at room temperature for 1 h. Membranes were washed three times for 5 min each with TBST and then incubated with the secondary antibody, anti-Rabbit IgG-peroxidase (Sigma) at a 1:5000 dilution in TBST-B for 1 h. Signals were detected by SuperSignal West Pico chemiluminescent substrate (Thermo Scientific) and visualized using a LAS-4000 (GE Healthcare). The densities of the SaeS protein bands were determined by quantification with ImageQuant software TL (GE Healthcare). To visualize equal loading, signals detected from non-specific binding of the SaeS antibody to an unknown cellular protein are shown. All Western blots were repeated at least three times with similar results.

### Coagulation Assay

To assess blood coagulating activity of the bacterial cells, overnight cultures of *S*. *aureus* strains were diluted 1:100 into fresh TSB and grown at 37°C for 4 h. Fifty microliters of bacterial culture were added to 0.5 ml of rehydrated BBL coagulase plasma with EDTA (rabbit, Becton Dickinson) in a sterile glass test tube and incubated at 37°C until a clot formed in the plasma. Coagulation time was recorded by observing the formation of a clot in the plasma as a function of time.

### Hemolysin Assay

Test strains were grown in TSB to exponential growth phase (OD_600_ = 1.0). One microliter of the cell suspension was spotted onto tryptic soy agar containing 5% rabbit blood (Becton Dickinson), and the plates were incubated at 37°C for 24 h. The hemolysis zones of colonies were imaged using a Canon ELPH-100HS camera (Canon), and the images were adjusted by Adobe Photoshop CS3 (Adobe). All hemolysin assays were performed in duplicate and repeated three times with similar results.

### Animal Test

NMΔ*sae* (pCL55-*saeRS*) strains carrying wild-type or mutant SaeS were grown in TSB to the exponential growth phase (OD_600_ = 1.0). Cells were washed in phosphate-buffered solution and suspended in PBS to OD_600_ = 0.4. The bacterial suspension (10^7^ cfu in 100 μl) was administered into 10 sex-matched 8 week-old Balb/c mice (Harlan) via retro-orbital injection. The infected mice were watched for 20 days. The survival curves were compared by Log-rank (Mantel-Cox) test with Prism 5 (GraphPad).

### Linker Peptide Sequence Comparison

Sequence comparison was carried out with the databases and the programs made available by the National Center for Biotechnology Information [40]. SaeS and GraS homologs were identified with PSI-BLAST search with the wild type SaeS and GraS protein sequences. Among the identified sequences, the ones with unusual features such as long linker peptides were removed by visual inspection, leaving 325 SaeS (96 from Staphylococcaceae, 136 from Streptococcaceae, 72 from Bacillaceae, 6 from Listeriaceae, 15 from other bacteria) and 330 GraS homolog sequences (116 from Staphylococcaceae, 203 from Bacillaceae, 11 from other bacteria). Sequence logos were prepared with WebLogo [41].

### Accession Numbers

*saeS* (NCBI-Gene ID:3913143); *saeSepi* (NCBI-Gene ID: 3241744); *saeR* (NCBI-Gene ID: 3913605); *graS* (NCBI-Gene ID: 3913958); *gfpopt* (GenBank: FJ169508.1); *phoB* (NCBI-Gene ID: 3914423).

## Supporting Information

S1 Fig Sequence comparison between SaeS from *S*. *aureus* and SaeS from *S*. *epidermidis*. Linker peptide sequence is indicated in red whereas transmembrane helix regions are shown in blue. TM1, transmembrane helix 1; TM2, transmembrane helix 2
(TIF)

S2 Fig A model for the high and low affinity targets of the SaeRS TCS. The positions of the basal and HNP1-induced kinase activities of SaeS in the graph are all hypothetical. However, the transcriptional patterns of the target genes are based on experimental observations.
(TIF)

S3 Fig SaeS_epi_ is not unstable. FLAG-tagged SaeS proteins were expressed and detected by Western blot analysis with either anti-SaeS antibody (SaeS) or anti-FLAG antibody (FLAG, Sigma-Aldrich). S_wt_, wild type SaeS; S_ELP_, SaeS with deletion of the linker peptide; S_epi_, SaeS of *S*. *epidermidis*.
(TIF)

S4 Fig The deletion mutants of SaeS, Δ9–31 and Δ41–63, are localized in the cell membrane. Cells were lysed and fractionated into cytoplasm and membrane fractions. SaeS was detected by Western blot analysis with anti-SaeS antibody.
(TIF)

S5 Fig The effect of the linker peptide mutations varies depending on the occupying amino acid. (A) Expression of the mutant SaeS proteins analyzed by Western blotting. The test strains were harvested at exponential growth phase; then an equal number of cells were used for the analysis. WT, wild type; VC, vector control. (B) The effect of the mutations in the linker peptide on the transcription of the low affinity target P*coa*. For induction of the SaeRS TCS, the cells were treated with HNP1 (5 μg/ml) for 2 h. All measurements were normalized by OD_600_. The error bars represent the standard error of the mean. AU, arbitrary unit. (C) The effect of the mutations in the linker peptide on the transcription of the high affinity target P*hla*. (D) The effect of the M31C mutation on the transcription of P*coa*. (E) The effect of the M31C mutation on the transcription of P*hla*.
(TIF)

S6 Fig Sequence analysis of the linker peptide region in SaeS and GraS homologs. These sequence logos display the relative frequency and information content at each position from 25–45 in a collection of aligned amino acid sequences with SaeS and GraS homologs. (A) Alignment of 325 SaeS homologs. (B) Alignment of SaeS homologs from Staphylococcaceae. (C) Alignment with SaeS homologs from Streptococcaceae. (D) Alignment of SaeS homologs from Bacillaceae. (E) Alignment of SaeS homologs from Listeriaceae. (F) Alignment of 330 GraS homologs. The number in parenthesis represents the number of the protein sequences used for the alignment. Amino acid numbering is according to SaeS or GraS sequence in *S*. *aureus* USA300. Red, positively charged amino acid; blue, negatively charged amino acid; green, polar amino acid; black, non-polar amino acid; orange, aromatic amino acid. x, non-conserved amino acid residue.
(TIF)

S1 Table Bacterial strains and plasmids used in this study. (DOCX)

S2 Table Oligonucleotides used in this study. (DOCX)
