# Supplementary figures and images for: The Extracytoplasmic Linker Peptide of the Sensor Protein SaeS Tunes the Kinase Activity Required for Staphylococcal Virulence in Response to Host Signals
Source: PLoS Pathog. 2015 Apr 7;11(4):e1004799. doi: 10.1371/journal.ppat.1004799 (PMC4388633; doi:10.1371/journal.ppat.1004799)

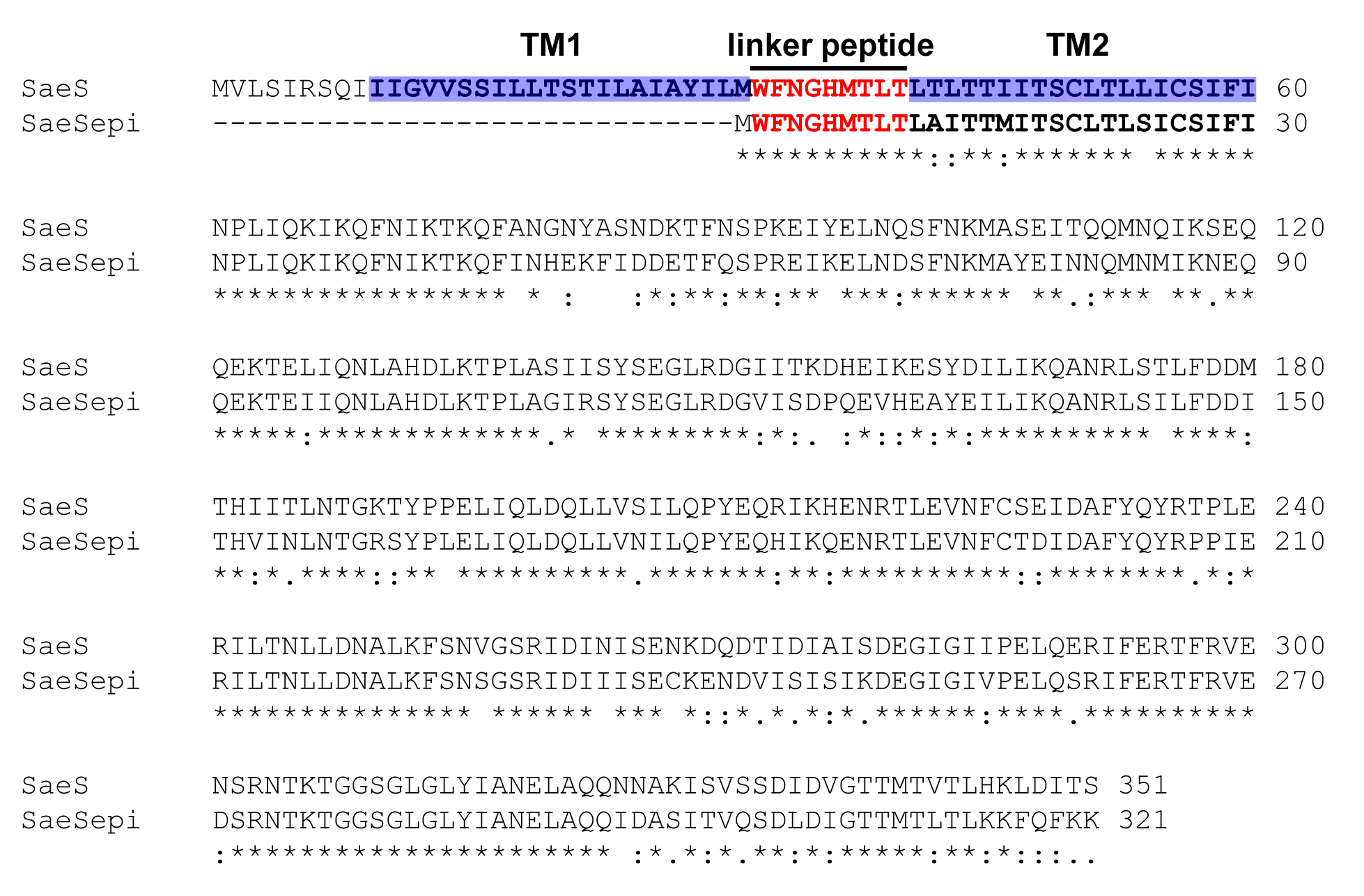

Supplement: S1 Fig — Linker peptide sequence is indicated in red whereas transmembrane helix regions are shown in blue. TM1, transmembrane helix 1; TM2, transmembrane helix 2 (TIF) [file ppat.1004799.s001.tif]

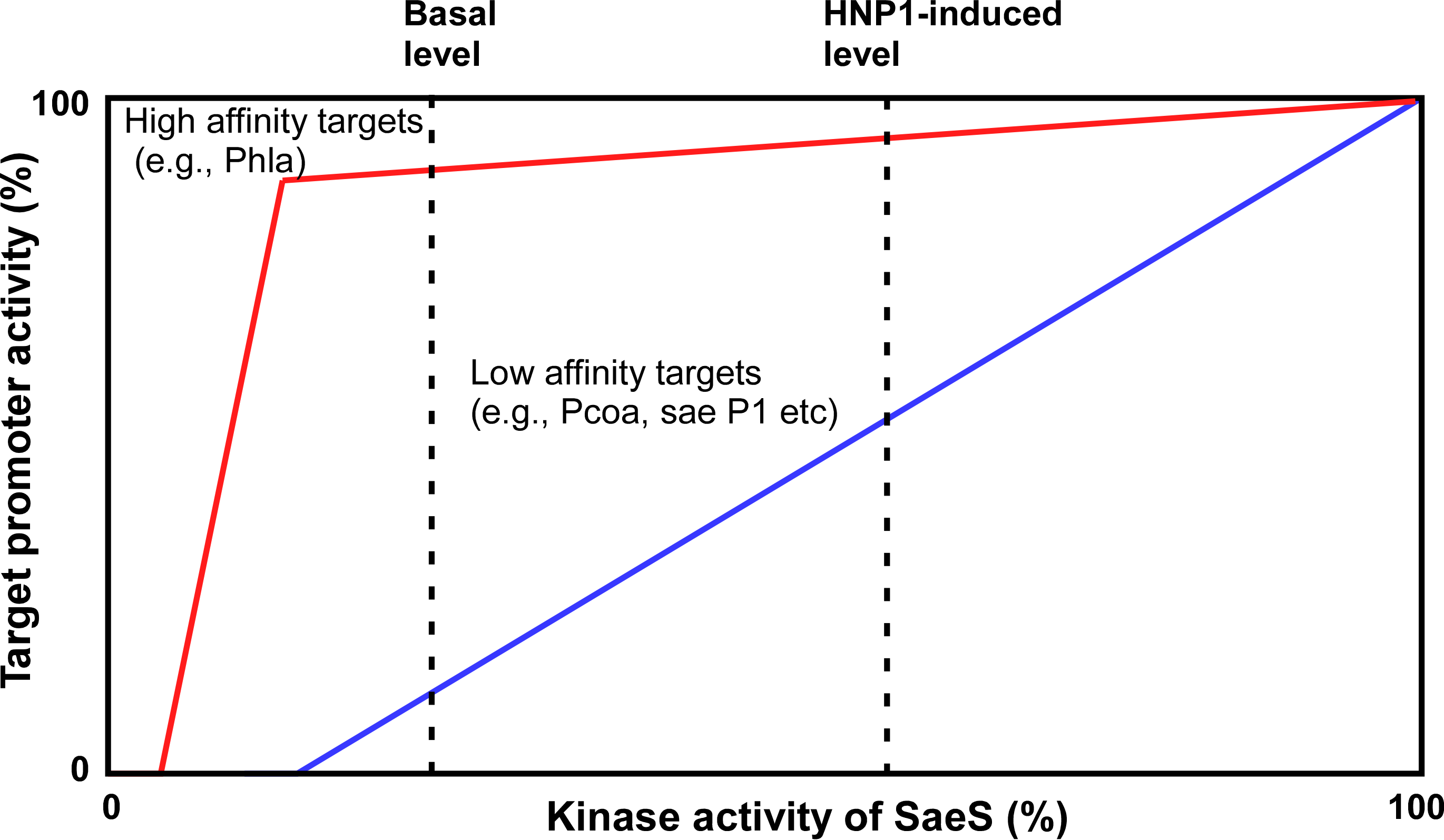

Supplement: S2 Fig — The positions of the basal and HNP1-induced kinase activities of SaeS in the graph are all hypothetical. However, the transcriptional patterns of the target genes are based on experimental observations. (TIF) [file ppat.1004799.s002.tif]

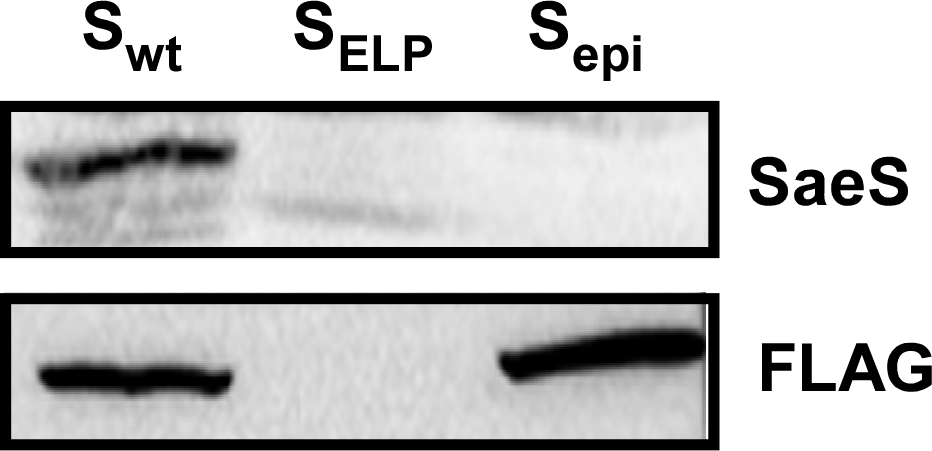

Supplement: S3 Fig — FLAG-tagged SaeS proteins were expressed and detected by Western blot analysis with either anti-SaeS antibody (SaeS) or anti-FLAG antibody (FLAG, Sigma-Aldrich). Swt, wild type SaeS; SELP, SaeS with deletion of the linker peptide; Sepi, SaeS of S. epidermidis. (TIF) [file ppat.1004799.s003.tif]

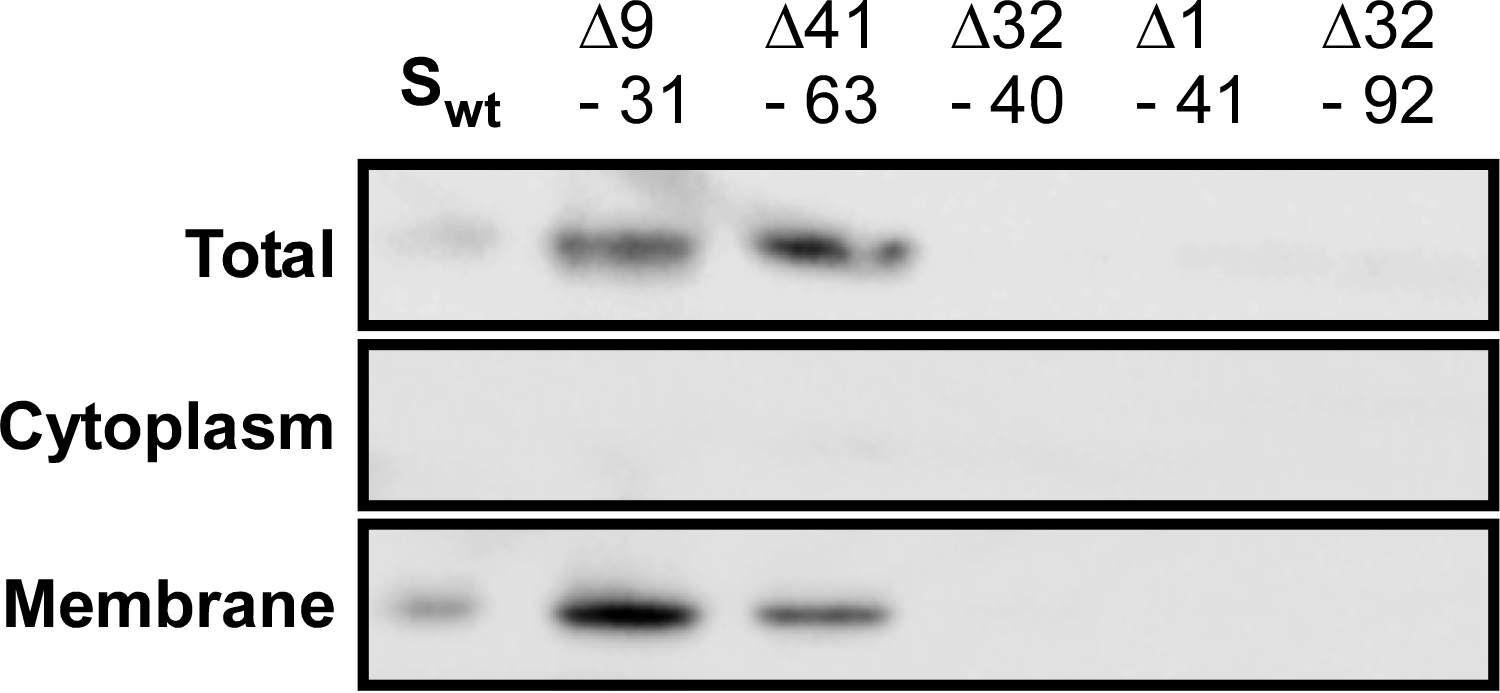

Supplement: S4 Fig — Cells were lysed and fractionated into cytoplasm and membrane fractions. SaeS was detected by Western blot analysis with anti-SaeS antibody. (TIF) [file ppat.1004799.s004.tif]

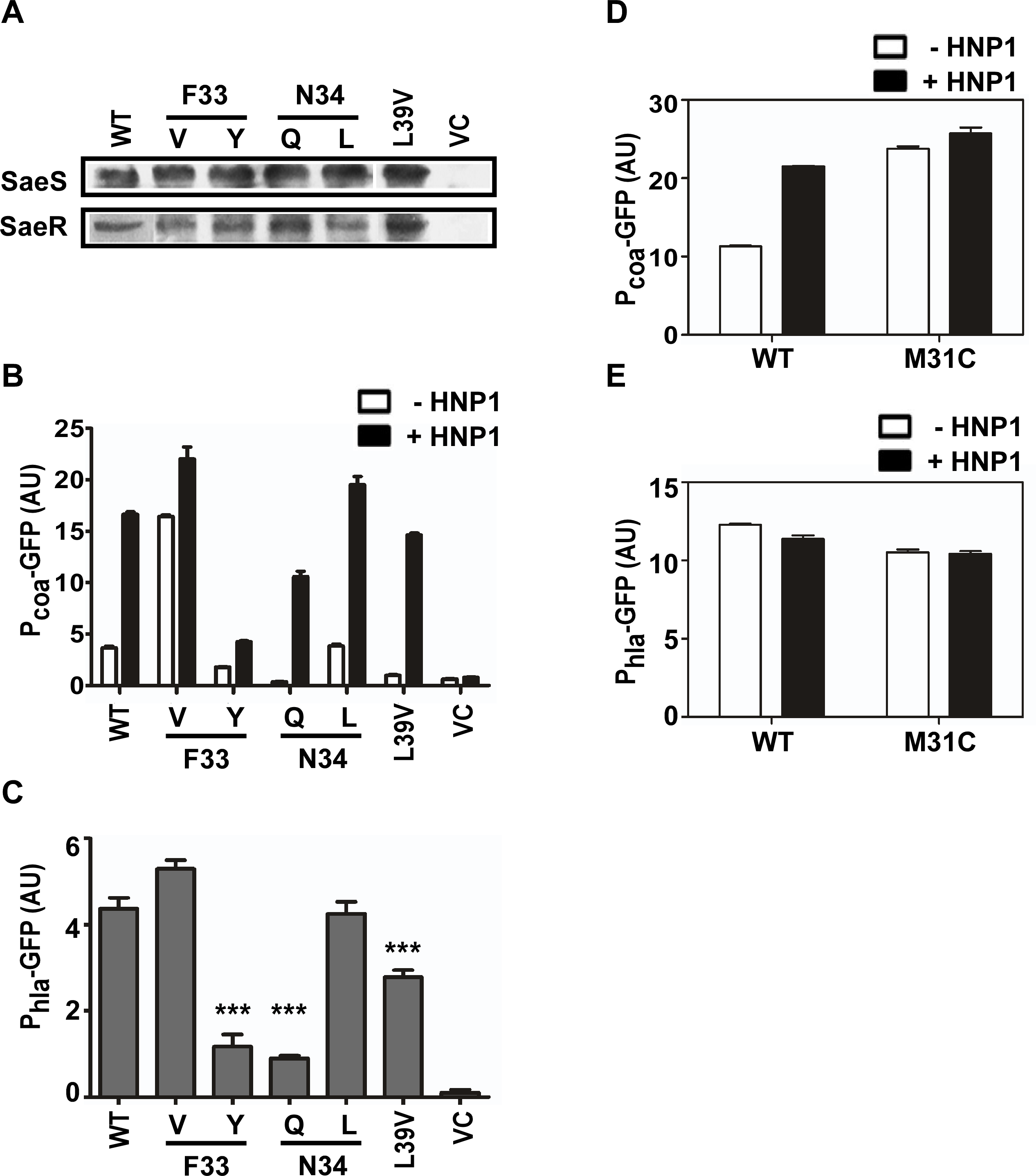

Supplement: S5 Fig — (A) Expression of the mutant SaeS proteins analyzed by Western blotting. The test strains were harvested at exponential growth phase; then an equal number of cells were used for the analysis. WT, wild type; VC, vector control. (B) The effect of the mutations in the linker peptide on the transcription of the low affinity target Pcoa. For induction of the SaeRS TCS, the cells were treated with HNP1 (5 μg/ml) for 2 h. All measurements were normalized by OD600. The error bars represent the standard error of the mean. AU, arbitrary unit. (C) The effect of the mutations in the linker peptide on the transcription of the high affinity target Phla. (D) The effect of the M31C mutation on the transcription of Pcoa. (E) The effect of the M31C mutation on the transcription of Phla. (TIF) [file ppat.1004799.s005.tif]

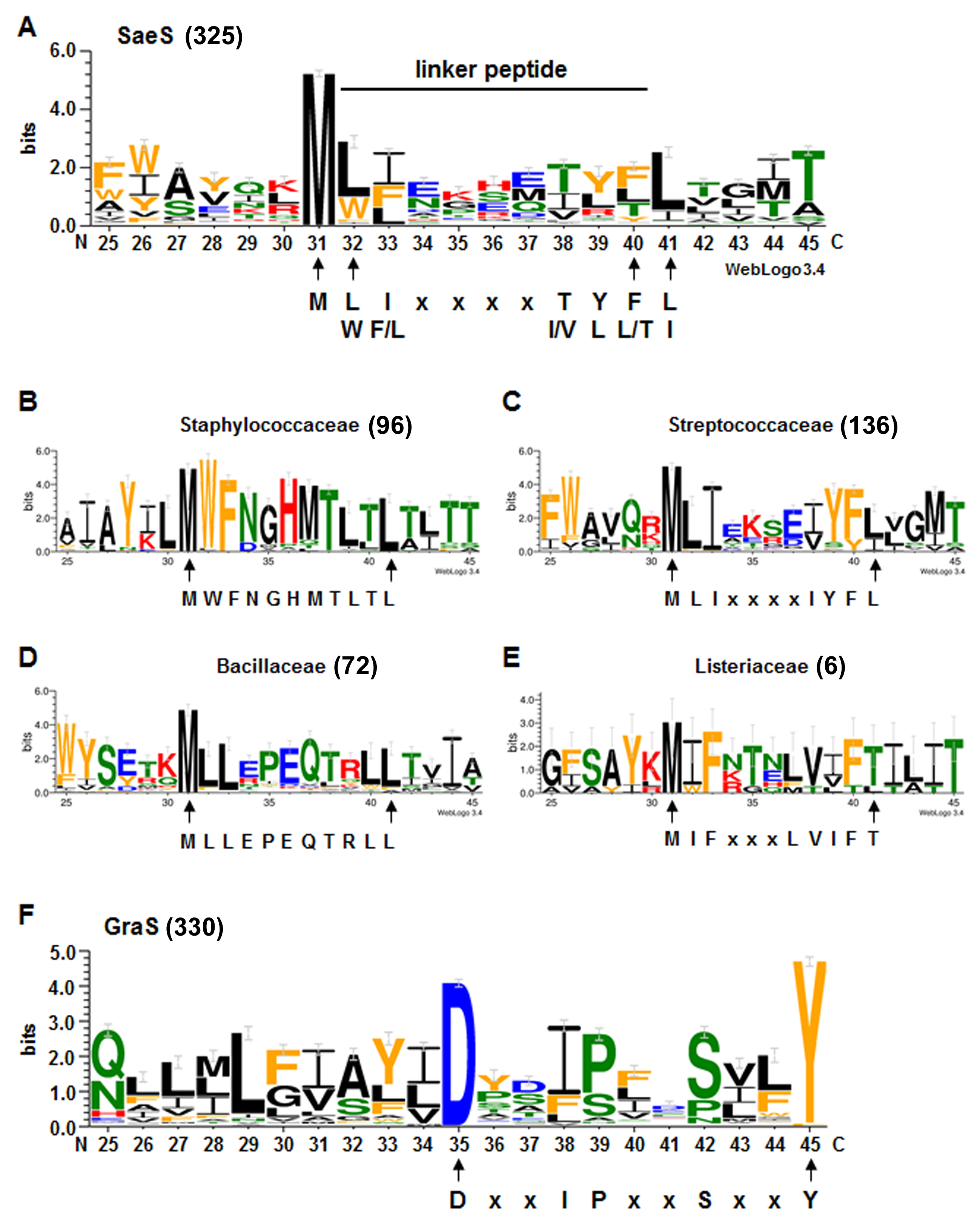

Supplement: S6 Fig — These sequence logos display the relative frequency and information content at each position from 25–45 in a collection of aligned amino acid sequences with SaeS and GraS homologs. (A) Alignment of 325 SaeS homologs. (B) Alignment of SaeS homologs from Staphylococcaceae. (C) Alignment with SaeS homologs from Streptococcaceae. (D) Alignment of SaeS homologs from Bacillaceae. (E) Alignment of SaeS homologs from Listeriaceae. (F) Alignment of 330 GraS homologs. The number in parenthesis represents the number of the protein sequences used for the alignment. Amino acid numbering is according to SaeS or GraS sequence in S. aureus USA300. Red, positively charged amino acid; blue, negatively charged amino acid; green, polar amino acid; black, non-polar amino acid; orange, aromatic amino acid. x, non-conserved amino acid residue. (TIF) [file ppat.1004799.s006.tif]
