# Supplementary material for: The Extracytoplasmic Linker Peptide of the Sensor Protein SaeS Tunes the Kinase Activity Required for Staphylococcal Virulence in Response to Host Signals
Source: PLoS Pathog. 2015 Apr 7;11(4):e1004799. doi: 10.1371/journal.ppat.1004799 (PMC4388633; doi:10.1371/journal.ppat.1004799)
Supplement: S1 Table — (DOCX) [file ppat.1004799.s007.docx]

**S1 Table. Bacterial strains and plasmids** **used in this study.**

| **Strain or plasmid** | **Relevant characteristic** | **Origin or reference** |
| --- | --- | --- |
| *E. coli* |  |  |
| DH5α | Plasmid free, restriction deficient | New England Biolabs |
|  |  |  |
| *S. aureus* |  |  |
| RN4220 | Restriction deficient, prophage cured | [[1](#_ENREF_1)] |
| Newman | Clinical isolate, L18P substitution in SaeS | [[2](#_ENREF_2)] |
| NMΔ*sae* | Newman with deletion of the *sae*operon | [[3](#_ENREF_3)] |
| NMΔ*phoB* | Newman with deletion of the *phoB*operon | This study |
|  |  |  |
| Plasmid |  |  |
| pIMAY | Allelic replacement plasmid | [[4](#_ENREF_4)] |
| pYJ335 | A shuttle vector for Gram - and Gram +, Erm^r^ | [[5](#_ENREF_5)] |
| pYJ-*gfp* | pYJ335 carrying a promoterless *gfp* | This study |
| pYJ335-P*coa*-*gfp* | pYJ335-GFP carrying *coa* promoter | This study |
| pYJ335-P*hla*-*gfp* | pYJ335-GFP carrying *hla* promoter | This study |
| pCL55 | A shuttle vector for Gram - and Gram +, Chl^r^ | [[6](#_ENREF_6)] |
| pCL55-*saeRS* | pCL55 carrying SaeR and SaeS | [[7](#_ENREF_7)] |
| pQL127 | pCL55-*saeRS* R6-*phoB* | This study |
| pQL106 | pCL55-*saeRS* G35-*phoB* | This study |
| pQL128 | pCL55-*saeRS* N71-*phoB* | This study |
| pQL20 | pCL55 carrying *saeR* and *saeSc* | This study |
| pQL52 | pCL55 carrying *saeR* and *saeS_ELP_* | This study |
| pQL91 | pCL55 carrying *saeR* and *saeS_ND_* | This study |
| pQL147 | pCL55 carrying *saeR* and *saeS_TM_* | This study |
| pQL110 | pCL55 carrying *saeR* and *saeS_epi_* | This study |
| pQL111 | pCL55 carrying *saeR* and *saeS_e-ND_* | This study |
| pQL73 | pCL55 carrying *saeR* and *saeS* W32A | This study |
| pQL74 | pCL55 carrying *saeR* and *saeS* F33A | This study |
| pQL75 | pCL55 carrying *saeR* and *saeS* N34A | This study |
| pQL76 | pCL55 carrying *saeR* and *saeS* G35A | This study |
| pQL77 | pCL55 carrying *saeR* and *saeS* H36A | This study |
| pQL78 | pCL55 carrying *saeR* and *saeS* M37A | This study |
| pQL79 | pCL55 carrying *saeR* and *saeS* T38A | This study |
| pQL80 | pCL55 carrying *saeR* and *saeS* L39A | This study |
| pQL81 | pCL55 carrying *saeR* and *saeS* T40A | This study |
| pQL148 | pCL55 carrying *saeR* and *saeS* G35V | This study |
| pQL149 | pCL55 carrying *saeR* and *saeS* G35L | This study |
| pQL150 | pCL55 carrying *saeR* and *saeS* G35I | This study |
| pQL151 | pCL55 carrying *saeR* and *saeS* G35F | This study |
| pQL152 | pCL55 carrying *saeR* and *saeS* G35P | This study |
| pQL153 | pCL55 carrying *saeR* and *saeS* G35W | This study |
| pQL154 | pCL55 carrying *saeR* and *saeS* G35S | This study |
| pQL155 | pCL55 carrying *saeR* and *saeS* G35C | This study |
| pQL156 | pCL55 carrying *saeR* and *saeS* G35M | This study |
| pQL157 | pCL55 carrying *saeR* and *saeS* G35N | This study |
| pQL158 | pCL55 carrying *saeR* and *saeS* G35Q | This study |
| pQL159 | pCL55 carrying *saeR* and *saeS* G35T | This study |
| pQL160 | pCL55 carrying *saeR* and *saeS* G35Y | This study |
| pQL161 | pCL55 carrying *saeR* and *saeS* G35E | This study |
| pQL162 | pCL55 carrying *saeR* and *saeS* G35D | This study |
| pQL163 | pCL55 carrying *saeR* and *saeS* G35K | This study |
| pQL164 | pCL55 carrying *saeR* and *saeS* G35R | This study |
| pQL165 | pCL55 carrying *saeR* and *saeS* G35H | This study |
| pQL182 | pCL55 carrying *saeR* and *saeS* F33V | This study |
| pQL183 | pCL55 carrying *saeR* and *saeS* F33Y | This study |
| pQL184 | pCL55 carrying *saeR* and *saeS* N34Q | This study |
| pQL185 | pCL55 carrying *saeR* and *saeS* N34L | This study |
| pQL188 | pCL55 carrying *saeR* and *saeS* L39V | This study |
| pQL221 | pCL55 carrying *saeR* and *saeS* M31C | This study |
| pRJ40 | pCL55 carrying *saeR* and *saeS* FLAG | This study |
| pRJ41 | pCL55 carrying *saeR* and *saeS_ELP_* FLAG | This study |
| pRJ42 | pCL55 carrying *saeR* and *saeS_epi_* FLAG | This study |

Am^r^,ampicillin resistance; Erm^r^, erythromycin resistance; Chl^r^, Chloramphenicol resistance

1. Kreiswirth BN, Lofdahl S, Betley MJ, O'Reilly M, Schlievert PM, et al. (1983) The toxic shock syndrome exotoxin structural gene is not detectably transmitted by a prophage. Nature 305: 709-712.

2. Duthie ES, Lorenz LL (1952) Staphylococcal coagulase; mode of action and antigenicity. J Gen Microbiol 6: 95-107.

3. Sun F, Li C, Jeong D, Sohn C, He C, et al. (2010) In the *Staphylococcus aureus* two-component system sae, the response regulator SaeR binds to a direct repeat sequence and DNA binding requires phosphorylation by the sensor kinase SaeS. J Bacteriol 192: 2111-2127.

4. Monk IR, Shah IM, Xu M, Tan MW, Foster TJ (2012) Transforming the Untransformable: Application of Direct Transformation To Manipulate Genetically *Staphylococcus aureus* and *Staphylococcus epidermidis*. MBio 3.

5. Ji Y, Marra A, Rosenberg M, Woodnutt G (1999) Regulated antisense RNA eliminates alpha-toxin virulence in *Staphylococcus aureus* infection. J Bacteriol 181: 6585-6590.

6. Lee CY, Buranen SL, Ye ZH (1991) Construction of single-copy integration vectors for *Staphylococcus aureus*. Gene 103: 101-105.

7. Jeong DW, Cho H, Lee H, Li C, Garza J, et al. (2011) Identification of the P3 promoter and distinct roles of the two promoters of the SaeRS two-component system in *Staphylococcus aureus.* J Bacteriol 193: 4672-4684.
