# Supplementary material for: The Extracytoplasmic Linker Peptide of the Sensor Protein SaeS Tunes the Kinase Activity Required for Staphylococcal Virulence in Response to Host Signals
Source: PLoS Pathog. 2015 Apr 7;11(4):e1004799. doi: 10.1371/journal.ppat.1004799 (PMC4388633; doi:10.1371/journal.ppat.1004799)
Supplement: S2 Table — (DOCX) [file ppat.1004799.s008.docx]

**S2 Table. Oligonucleotides used in this study.**

| **Name** | **Sequence (5’ to 3’)** | | **Product** | |  |
| --- | --- | --- | --- | --- | --- |
| **SaeS-PhoB fusions (V, vector; I, Insert)** | | | | |  |
| P1986 | | ATTGGATTGGAAGTAC GCTTTGGCAGTTTATTCTTGACATGTA | | pIMAY, V | |
| P1987 | | ATTGGAAGTGGATAAC CGAAGTGATCTTCCGTCACAGGTATT | | pIMAY, V | |
| P2002 | | TACTTCCAATCCAATG TGCATTCACCCTATACTTTATATC | | *ΔphoB*, I | |
| P2003 | | AAGACATCCTCCTGAGTATATG | | *ΔphoB*, I | |
| P2004 | | CATATACTCAGGAGGATGTCTT AGCATAAAAAAATTCCCGAGCAC | | *ΔphoB*, I | |
| P2005 | | TTATCCACTTCCAATG ATTATGCATATTGGGATCAAGCG | | *ΔphoB*, I | |
| P2162 | | AGTCAAATCATTATTGGCGTC | | R6-PhoB, V | |
| P2163 | | TCTAATTGATAACACCATTATCG | | R6-PhoB, V | |
| P2164 | | CGATAATGGTGTTATCAATTAGAATGCAATCCGATAAAAGTTCTAAAG | | R6-PhoB, I | |
| P2165 | | GACGCCAATAATGATTTGACT CTTGAATATATCAAATATTATTTTTGC | | R6-PhoB, I | |
| P2103 | | CATATGACACTAACTTTGACC | | G35-PhoB, V | |
| P2104 | | GCCGTTAAACCACATTAAAATAT | | G35-PhoB, V | |
| P2107 | | ATATTTTAATGTGGTTTAACGGC ATGCAATCCGATAAAAGTTCTAAAG | | G35-PhoB, I | |
| P2108 | | GGTCAAAGTTAGTGTCATATG CTTGAATATATCAAATATTATTTTTGC | | G35-PhoB, I | |
| P2166 | | ATAAAAACTAAGCAATTTGCTAAC | | N71-PhoB, V | |
| P2167 | | ATTAAACTGCTTAATTTTTTGTATAAG | | N71-PhoB, V | |
| P2168 | | CTTATACAAAAAATTAAGCAGTTTAATATGCAATCCGATAAAAGTTCTAAAG | | N71-PhoB, I | |
| P2169 | | GTTAGCAAATTGCTTAGTTTTTATCTTGAATATATCAAATATTATTTTTGC | | N71-PhoB, I | |
| **Deletion and hybrid mutants of SaeS (V, vector)** | | | | | |
| P1887 | | ATGAAAGAAATTTATGAATTAAATCAATC | | SaeSc | |
| P1888 | | TTATCGGCTCCTTTCAAATTTATATC | | SaeSc | |
| P1958 | | CATTAAAATATATGCAATTGCTAAAA | | Δ32-40 | |
| P1960 | | AGTTAGTGTCATATGGCCGTTA | | Δ32-40 | |
| P2226 | | CTTATGTTAGGCATTTGGTTTAACGGCCATATGACACTAACTGACAGTTTATT | | SaeS_TM_ | |
| P2227 | | GAAGTTTAAAAACAATATCCAAAAT | | SaeS_TM_ | |
| P1961 | | TTGACCTTAACGACAATAATTAC | | SaeS_ELP_ | |
| P1962 | | GCATATATTTTAATGGATTATGATTTTCCAATAGACAGTTTA TTGACCTTAAC | | SaeS_ELP_ | |
| P2059 | | ATACAAAAAATTAAGCAGTTTAATAT | | SaeS_ND,_ V | |
| P2060 | | TATCGGCTCCTTTCAAATTTATATC | | SaeS_ND’_ V | |
| P2061 | | GATATAAATTTGAAAGGAGCCGATAATGAATAATTTGAAATGGGTAGC | | SaeS_ND_ | |
| P2062 | | ATATTAAACTGCTTAATTTTTTGTATAAAATATGTCAATAAAAGAAAAATCATTG | | SaeS_ND_ | |
| P2141 | | TCCGATTTATTTATAAAATAAAATGC | | SaeS_epi_, V | |
| P2142 | | TATCGGCTCCTTTCAAATTTATATC | | SaeS_epi_ and SaeS_e-ND_, V | |
| P2143 | | GATATAAATTTGAAAGGAGCCGATAATGTGGTTTAATGGTCACATGAC | | SaeS_epi_ | |
| P2144 | | GCATTTTATTTTATAAATAAATCGGATTACTTTTTAAATTGAAATTTTTTTAAG | | SaeS_epi_ | |
| P2145 | | GATATAAATTTGAAAGGAGCCGATAATGGTGTTATCAATTAGAAGTC | | SaeS_e-ND_ | |
| P2146 | | TATTGAATTGCTTAATCTTCTGAATAAGTGGATTAATAAAAATACTAC | | SaeS_e-ND_ | |
| P2147 | | ATTCAGAAGATTAAGCAATTCAATA | | SaeS_e-ND_, V | |
| **Single substitution mutants of SaeS** | | | | |  |
| P2568 | ATTGCATATATTTTA tgc TGGTTTAACGGCCAT | | M31C | |  |
| P2569 | ATGGCCGTTAAACCAGCATAAAATATATGCAAT | | M31C | |  |
| P2012 | ATATTTTAATG GCA TTTAACGGCC | | W32A | |  |
| P2013 | GGCCGTTAAA TGC CATTAAAATAT | | W32A | |  |
| P2016 | CATATATTTTAATGTGG GCA AACGGCCATATG | | F33A | |  |
| P2017 | CATATGGCCGTTTGCCCACATTAAAATATATG | | F33A | |  |
| P2018 | CATATATTTTAATGTGGTTT gca GGCCATATGACAC | | N34A | |  |
| P2019 | GTGTCATATGGCCTGCAAACCACATTAAAATATATG | | N34A | |  |
| P2020 | TAATGTGGTTTAAC gca CATATGACACTAACTTTG | | G35A | |  |
| P2021 | CAAAGTTAGTGTCATATG TGC GTTAAACCACATTA | | G35A | |  |
| P2057 | ATTTTAATGTGGTTTAACGGCgcaATGACACTAACTTTGACCTTAAC | | H36A | |  |
| P2058 | GTTAAGGTCAAAGTTAGTGTCATTGCGCCGTTAAACCACATTAAAAT | | H36A | |  |
| P2024 | GGTTTAACGGCCAT gca ACACTAACTTTGACC | | M37A | |  |
| P2025 | GGTCAAAGTTAGTGTTGCATGGCCGTTAAACC | | M37A | |  |
| P2026 | GTTTAACGGCCATATG gca CTAACTTTGACCTTAAC | | T38A | |  |
| P2027 | GTTAAGGTCAAAGTTAGTGCCATATGGCCGTTAAAC | | T38A | |  |
| P2028 | CGGCCATATGACA gca ACTTTGACCTTAACG | | L39A | |  |
| P2029 | CGTTAAGGTCAAAGTTGCTGTCATATGGCCG | | L39A | |  |
| P2030 | GGCCATATGACACTA gca TTGACCTTAACGAC | | T40A | |  |
| P2031 | GTCGTTAAGGTCAATGCTAGTGTCATATGGCC | | T40A | |  |
| P2228 | TAATGTGGTTTAAC gtg CATATGACACTAACTTTG | | G35V | |  |
| P2229 | CAAAGTTAGTGTCATATG CAC GTTAAACCACATTA | | G35V | |  |
| P2230 | TAATGTGGTTTAAC cta CATATGACACTAACTTTG | | G35L | |  |
| P2231 | CAAAGTTAGTGTCATATG TAG GTTAAACCACATTA | | G35L | |  |
| P2232 | TAATGTGGTTTAAC atc CATATGACACTAACTTTG | | G35I | |  |
| P2233 | CAAAGTTAGTGTCATATG GAT GTTAAACCACATTA | | G35I | |  |
| P2234 | TAATGTGGTTTAAC ttc CATATGACACTAACTTTG | | G35F | |  |
| P2235 | CAAAGTTAGTGTCATATG GAA GTTAAACCACATTA | | G35F | |  |
| P2236 | TAATGTGGTTTAAC cca CATATGACACTAACTTTG | | G35P | |  |
| P2237 | CAAAGTTAGTGTCATATG TGG GTTAAACCACATTA | | G35P | |  |
| P2242 | TAATGTGGTTTAAC tgg CATATGACACTAACTTTG | | G35W | |  |
| P2243 | CAAAGTTAGTGTCATATG CCA GTTAAACCACATTA | | G35W | |  |
| P2244 | TAATGTGGTTTAAC tcg CATATGACACTAACTTTG | | G35S | |  |
| P2245 | CAAAGTTAGTGTCATATG CGA GTTAAACCACATTA | | G35S | |  |
| P2246 | TAATGTGGTTTAAC tgc CATATGACACTAACTTTG | | G35C | |  |
| P2247 | CAAAGTTAGTGTCATATG GCA GTTAAACCACATTA | | G35C | |  |
| P2248 | TAATGTGGTTTAAC atg CATATGACACTAACTTTG | | G35M | |  |
| P2249 | CAAAGTTAGTGTCATATG CAT GTTAAACCACATTA | | G35M | |  |
| P2250 | TAATGTGGTTTAAC aac CATATGACACTAACTTTG | | G35N | |  |
| P2251 | CAAAGTTAGTGTCATATG GTT GTTAAACCACATTA | | G35N | |  |
| P2252 | TAATGTGGTTTAAC cag CATATGACACTAACTTTG | | G35Q | |  |
| P2253 | CAAAGTTAGTGTCATATG CTG GTTAAACCACATTA | | G35Q | |  |
| P2254 | TAATGTGGTTTAAC acg CATATGACACTAACTTTG | | G35T | |  |
| P2255 | CAAAGTTAGTGTCATATG CGT GTTAAACCACATTA | | G35T | |  |
| P2256 | TAATGTGGTTTAAC tac CATATGACACTAACTTTG | | G35Y | |  |
| P2257 | CAAAGTTAGTGTCATATG GTA GTTAAACCACATTA | | G35Y | |  |
| P2238 | TAATGTGGTTTAAC gag CATATGACACTAACTTTG | | G35E | |  |
| P2239 | CAAAGTTAGTGTCATATG CTC GTTAAACCACATTA | | G35E | |  |
| P2240 | TAATGTGGTTTAAC gac CATATGACACTAACTTTG | | G35D | |  |
| P2241 | CAAAGTTAGTGTCATATG GTC GTTAAACCACATTA | | G35D | |  |
| P2258 | TAATGTGGTTTAAC aag CATATGACACTAACTTTG | | G35K | |  |
| P2259 | CAAAGTTAGTGTCATATG CTT GTTAAACCACATTA | | G35K | |  |
| P2260 | TAATGTGGTTTAAC cgc CATATGACACTAACTTTG | | G35R | |  |
| P2261 | CAAAGTTAGTGTCATATG GCG GTTAAACCACATTA | | G35R | |  |
| P2262 | TAATGTGGTTTAAC cac CATATGACACTAACTTTG | | G35H | |  |
| P2263 | CAAAGTTAGTGTCATATG GTG GTTAAACCACATTA | | G35H | |  |
| P2337 | CATATATTTTAATGTGG gtg AACGGCCATATG | | F33V | |  |
| P2338 | CATATGGCCGTTCACCCACATTAAAATATATG | | F33V | |  |
| P2339 | CATATATTTTAATGTGG tac AACGGCCATATG | | F33Y | |  |
| P2340 | CATATGGCCGTTGTACCACATTAAAATATATG | | F33Y | |  |
| P2341 | CATATATTTTAATGTGGTTT cag GGCCATATGACAC | | N34Q | |  |
| P2342 | GTGTCATATGGCCCTGAAACCACATTAAAATATATG | | N34Q | |  |
| P2343 | CATATATTTTAATGTGGTTT cta GGCCATATGACAC | | N34L | |  |
| P2344 | GTGTCATATGGCCTAGAAACCACATTAAAATATATG | | N34L | |  |
| P2349 | CGGCCATATGACA gtg ACTTTGACCTTAACG | | L39V | |  |
| P2350 | CGTTAAGGTCAAAGTCACTGTCATATGGCCG | | L39V | |  |
| P2056 | TACTTCCAATCCAATG gagtggtataagtggtttttcg | | *saeS* | |  |
| P2015 | TTATCCACTTCCAATG atgatgagaaggatacccataaag | | *saeS* | |  |
| **Promoter-*gfp* fusions** | | | | |  |
| P1969 | GGGGTACCATTGGAAGTGGATAACATGTCAAAAGGAGAAGAATTATTTAC | | pYJ-*gfp* | |  |
| P1970 | CCG CTCGAG TTACTTATATAATTCATCCATTCCGT | | *gfp* | |  |
| P1747 | ATTGGATTGGAAGTAC GGTACCGAGCTCGAATTCACTG | | pYJ-*gfp* | |  |
| P1973 | TACTTCCAATCCAATG AATTGAATTGTAAATACTTTCTAATC | | P*coa* | |  |
| P1974 | TTATCCACTTCCAATG AATTTTTTAATTCCTCCAAAATGTAATTG | | P*coa* | |  |
| P1992 | TTATCCACTTCCAATG ATTACAATATAAAAATACAAATATCTTAG | | P*hla* | |  |
| P1993 | TACTTCCAATCCAATG TTAATATATAGTTAATTTTTATTTAATAG | | P*hla* | |  |
| **SaeS-FLAG fusions** | | | | |  |
| P1729 | ATTGGAAGTGGATAAC GAATTCTTGAAGACGAAAGGGCCTCG | | pCL55 | |  |
| P1859 | ATTGGATTGGAAGTAC GGATCCGGAGGGATGTAAAATG | | pCL55 | |  |
| P167 | TTATCCACTTCCAATG GAGTGGTATAAGTGGTTTTTCG | | All SaeS | |  |
| P365 | TACTTCCAATCCAATGttacttatcgtcgtcatccttgtaatcTGACGTAATGTCTAATTTGTGTAATG | | SaeS & SaeS_ELP_ | |  |
| P366 | TACTTCCAATCCAATGttacttatcgtcgtcatccttgtaatcCTTTTTAAATTGAAATTTTTTTAAGG | | SaeS_epi_ | |  |
